# Facile synthesis of anthranilic acid based dual functionalized novel hyper cross-linked polymer for promising CO_2_ capture and efficient Cr^3+^ adsorption

**DOI:** 10.1038/s41598-024-61584-4

**Published:** 2024-05-17

**Authors:** Amin Abid, Saqlain Raza, Ahmad Kaleem Qureshi, Sajjad Ali, Isham Areej, Shahid Nazeer, Bien Tan, Wedad A. Al-onazi, Muhammad Rizwan, Rashid Iqbal

**Affiliations:** 1 Department of Chemistry, University of Sahiwal, Sahiwal, 57000 Pakistan; 2 Faculty of Agriculture and Environment, The Islamia University of Bahawalpur, Bahawalpur, 63100 Pakistan; 3 Huazhong University of Science and Technology (HUST), Wuhan, China; 4 Department of Chemistry, College of Science, King Saud University, P.O. 22452, 11495 Riyadh, Saudi Arabia; 5 Institute of Crops Science and Resource Conservation (INRES), University of Bonn, Bonn, Germany

**Keywords:** Polymers, Adsorption, Heavy metals, Carbon dioxide, Chromium, Environmental sciences, Chemistry, Materials science

## Abstract

A novel hyper cross-linked polymer of 2-Aminobenzoic acid (HCP-AA) is synthesized for the adsorption of Cr^3+^ and CO_2_. The Brunauer–Emmett–Teller surface area of HCP-AA is 615 m^2^ g^−1^. HCP-AA of particle size 0.5 nm showed maximum adsorption of Cr^3+^ for lab prepared wastewater (93%) while it was 88% for real industrial wastewater. It is might be due to electrostatic interactions, cation-π interactions, lone pair interactions and cation exchange at pH 7; contact time of 8 min; adsorbent dose 0.8 g. The adsorption capacity was calculated 52.63 mg g^−1^ for chromium metal ions at optimum conditions. Freundlich isotherm studies R^2^ = 0.9273 value is the best fit and follows pseudo second order kinetic model (R^2^ = 0.979). The adsorption is found non-spontaneous and exothermic through thermodynamic calculations like Gibbs free energy (ΔG), enthalpy change (ΔH) and entropy change (ΔS) were 6.58 kJ mol^−1^, − 60.91 kJ mol^−1^ and − 45.79 kJ mol^−1^ K^−1^, respectively. The CO_2_ adsorption capacity of HCP-AA is 1.39 mmol/g with quantity of 31.1 cm^3^/g (6.1 wt%) at 273Kwhile at 298 K adsorption capacity is 1.12 mmol/g with quantity 25.2 cm^3^/g (5 wt%). Overall, study suggests that carboxyl (–COOH) and amino (–NH_2_) groups may be actively enhancing the adsorption capacity of HCP-AA for Cr^3+^ and CO_2_.

## Introduction

There is no life without clean drinking water and fresh air but due to industrialization and population bloom, clean sources of water are declining day by day and fresh air is polluted with primary and secondary air pollutants^1–3^. Despite its huge abundance everywhere, clean water is very limited as compared to its use^4,5^. Heavy metals have serious toxic effects and cause serious diseases like^6^ diarrhea, cardiovascular diseases, genotoxicity, lung diseases, cancer, damage to kidneys and bones, anemia, and eczema of skin etc^7–10^.

Porous polymers are now a days, extensively used because they have important scientific and day-today applications such as heavy metal uptake, gas adsorption, drug delivery system, photo catalysis, super capacitors and renewable energy sources due to their novel features such as high BET surface area, microporous structure, high thermal stability and availability of a variety of functional groups^11–13^. The porous polymers can be classified as macroporous having pores larger than 50 nm, mesoporous having pores between 2 and 50 nm and microporous having pores smaller than 2 nm. The hyper cross-linked polymer is a type of pure organic materials and it exhibits highly cross-linked morphology. The HCP was first developed by Davankov and Tsyrupa in 1970s^14^. They synthesized HCP of polystyrene by using post crosslinking of the linear polystyrene chains from Friedel Craft reaction^15–17^. The HCPs synthesis starts from several aromatic monomers such as benzene and derivatives of benzene^18^. The first HCP was based on linear polystyrene (HCL-PS) precursor^19–21^.

Now a day, adsorption is considered as one of the most efficient method for removal of heavy metals^18,22,23^. Hybrid porous materials such as HCPs are extensively applied as adsorbents because of their large pore volume, greater surface area, strong chemical and thermal stabilities, low cost and easy synthesis^24,25^. The efficiency of these adsorbents is measured by different factors such as stability, typical adsorption parameters as well as functional sites providing specific adsorbate and adsorbent interactions like hydrogen bonds, π–π interactions and electrostatic interactions etc. Due to these interactions, different polymers are used in field of medicine as drug delivery, catalysis^26^, and adsorption^27,28^.

Tan and co-workers presented a cost-effective technique towards the production of micro-porous polymers of different types by using aromatic building blocks^29,30^ for different applications such as, heterogeneous catalysis, semiconductors, luminescence, water treatment and gas storage etc. The highly porous polymer was synthesized by Friedel–Craft alkylation with self-condensation benzyl alcohol aromatic mono-hydroxyl-methyl compounds^29,31,32^. In past, activated carbon and zeolite were also used for adsorption and removal of metal ions but they showed low adsorption ability^19,33–39^.

James et al. synthesized HCP based on derivatives of sulphonated materials in which 4,4bischloromethyl 1,1 biphenyl was used as sulphonated material. New HCPs were synthesized and named as SHCP 1 and SHCP 2. The SHCP 1 was synthesized by metal free alkylation reaction. While, SHCP 2 was synthesized by Friedel Crafts reaction catalyzed by Lewis’s acid. These two polymers have large surface area of 500 m^2^ g^−1^. SHCP 1 showed excellent adsorption properties for ions of Sr and Cs in an aqueous solution. A maximum uptake was 95.6 mg g^−1^ for Sr and 273 mg g^−1^ for Cs. These polymers were also shows good adsorption properties for the adsorption of Na, K, Mg and Ca and follow the pseudo second order reactions^21,40^. A HCP of iminodiacetic acid (IDA-HCP) for water purification from heavy metals ions is prepared. The synthesized polymer exhibited excellent adsorption properties because of the presence of carboxylate and amino groups. These functional groups make IDA-HCP excellent candidate for the adsorption of different metals including Zn^+2^, Mg^+2^, Hg^+2^ and others^41,42^.

A hyper cross-linked nanometer-sized chelating agent, denoted as HCNSCR, was successfully synthesized and experimental findings unveiled that HCNSCR exhibited notable adsorption capacities for the targeted metal ions, with values of 1.2 mmol/g for Pb, 1.0 mmol/g for Cd, and 0.9 mmol/g for Zn. Furthermore, the optimal pH range for achieving the highest adsorption performance was identified to fall between 5.5 and 6.3. To describe the adsorption behavior more comprehensively, the researchers employed a Langmuir isotherm model; it has been found that it provided an adequate match to the adsorption data. In addition, a pseudo-second-order pattern was observed in the adsorption kinetics^37,38^. The analysis conducted by Podkoscielna et al. involved the modification of commercially available St DVB (styrene–divinylbenzene) resin by introducing thiol (SH) groups into the polymer. The thiol groups were incorporated into St DVB by treating it with H_2_SO_4_ followed by either SnCl_2_ or POCl_3_. The purpose of the modification was to investigate the ability of the St DVB SH material to remove heavy metal ions (Cu^+2^, Zn^+2^, Cd^+2^, Pb^+2^, and Ni^+2^) from aqueous solutions. The results indicated that the novel modified polymer exhibited adsorption behavior in accordance with the Langmuir and Freundlich isotherm models^37,43–45^.

The most confronting challenge for human is global warming. The reason behind is the rapid increase in carbon dioxide concentration in atmosphere due to anthropogenic activities^46^. The most facile way to eliminate it is carbon capture and its storage (CCS). Initiation of CCS is too expensive. In some industries and power plants, post combustion capture (PCC) technology is used due to its medium feasibility, less cost than CCS, and easy installation without major modifications. Recently, Monoethanolamine (MEA) scrubbing is set as standard for PCC technology but it has some drawbacks to power plants. It is corrosive as well as need high energy for rejuvenation^47,48^.

The research and development of novel technologies and materials, that selectively capture CO_2_, need growing attention because the classical method of adsorbing CO_2_ using amine solution has various drawbacks, including less economical regeneration and equipment corrosion. Zeolites and activated carbon are the primary solid adsorbents for CO_2_ capture. In contrast, porous materials are an alternate method of capturing CO_2_ and have gained a lot of attention in recent years. The main advantages of porous materials are affordability, convenience of use, high chemical and thermal stability, and strong adsorption of CO_2_^49^.

Unfortunately, due to strong affinity for H_2_O, MOFs, belong to porous crystalline materials; have limited use under high humidity. It is also difficult for these POPs to adsorb CO_2_ more than 30% wt% at 0 °C and 1.0 bar^50^. Fortunately, a large number of alternative organic porous materials have been thoroughly investigated for CO_2_ adsorption, including polymers of intrinsic micro porosity (PIMs), conjugated microporous polymers (CMPs), HCPs, and covalent organic frameworks (COFs). Porous carbons have demonstrated potential for CO_2_ adsorption because of their high specific surface area, large pore volume, and adjustable surface^51,52^.

The main features of a carbon dioxide adsorbent include high CO_2_ selectivity, greater CO_2_ adsorption capability, minimal heat of adsorption, good chemical endurance, significant thermal and mechanical durability, fabrication scalability, suitable morphology, low toxicity and cost affectivity. When choosing CO_2_ adsorbents, excellent CO_2_ acceptability at low pressures is a crucial factor^17^. Therefore, designing physical adsorbents with protic electronegative functions by the introduction of nitrogen groups like amine and amide is valuable fabrication with high CO_2_ selectivity^47,53^.

Using melamine and resorcinol, Bing et al. created a porous carbon that was doped with nitrogen. They discovered that as the nitrogen content increased, it increased the CO_2_ adsorption. At the temperature of 273 K and pressure 1.0 bar, Nandi et al. reported that porous N-doped activated carbon monoliths achieved remarkable CO_2_ adsorption of 506 mg/g^48,54^. Triazine had also been utilized as a building block in recent times to create HCPs with nitrogen in the backbone. Triazine-based HCPs are regarded as innovative materials for gas sorption research due to their exceptional CO_2_ selectivity over N_2_^55,56^.

In this study, the fabrication of 2-Aminobenzoic Acid based dual functionalized polymer (HCP-AA) by Friedel Crafts reaction is reported by using cost effective and less toxic cross-linker CCl_4_, this is the novelty of this research. Previously in literature mostly used cross-linkers were having toxicity as well as they are expensive like formaldehyde dimethyl acetal (FDA). Furthermore, the use of same adsorbent for air purification and water treatment is also a novel concept. Industrial wastewater were used to determine the efficiency of HCP-AA and got promising results. The synthesized HCP has microporous structure, which make it excellent candidate for adsorption studies. The adsorption capacity of HCP-AA for chromium metal ions and CO_2_ gas is determined. The adsorption capacity is 52.63 m g g^−1^ for chromium metal ions and 1.3 9 mmol for CO_2_ at optimum conditions. Moreover, this HCP-AA can be recycled with minimum decrease in adsorption capacity. The potent adsorption capacity of HCP-AA is due to its high surface area, abundant porosity, oxygen and nitrogen rich nature. While, this synthesis has some drawbacks as it is time taking as well as requires high consumption of energy. Our research group is working to resolve these problems.

## Materials and methods

### Materials

Anthranilic acid (monomer), Carbon tetrachloride (cross-linker), Dichloroethane (DCE) (solvent), Ferric chloride (catalyst), Ethanol and Chromium Chloride were obtained from Sigma Organics and used as received in their pure form.

### Fabrication of hyper cross-linked polymer of anthranilic acid (HCP-AA)

In a round bottom flask fitted with condenser carrying 10 mL of solvent (Dichloroethane), 0.3 g of aromatic monomer (anthranilic acid), 1 mL of cross-linker (CCl_4_) and 0.3 g of Ferric Chloride (FeCl_3_), used as a catalyst, were continuously mixed and heated in an oil bath. Initially, temperature was maintained at 45 °C for 4–5 h, and then raised to 80–85 °C for 19–20 h till product was formed. The thick paste like appearance indicates the syntheses of HCP-AA which was washed with ethanol till pure HCP-AA was obtained after the removal of excessive solvent and FeCl_3_ (washed until brown color of FeCl_3_ disappeared). After filtrations, purified product was collected in china dish and dried at 100 °C in an oven^57,58^.

### Equipment

FTIR spectra were acquired using a Thermo Nicolet Nexus 670 spectrophotometer. Elemental composition analysis of the hyper cross-linked polymers (HCPs) was conducted through Energy Dispersive X-ray Spectroscopy (EDX). Scanning Electron Microscope (SEM) imaging and analysis were performed using a Zeiss Ultra-55 instrument. Transmission Electron Microscope (TEM) analysis was conducted with a JEM-2100 plus microscope. X-ray Diffraction (XRD) powder analysis of the synthesized polymer samples was carried out using a Smart Lab TM 3 kW X-ray diffractometer. Thermogravimetric analysis was carried out by using Netzsch Jupiter thermal analyzer. To determine the BET surface area, pore volume, pore size and nitrogen adsorption/ desorption isotherms of the HCP-AA, Micro-metrics ASAP 2020 M and porosity analyzer from Micrometrics, USA were used.

## Results and discussion

### Characterization of HCP of anthranilic acid (HCP-AA)

#### Physical appearance

**Figure 1:**
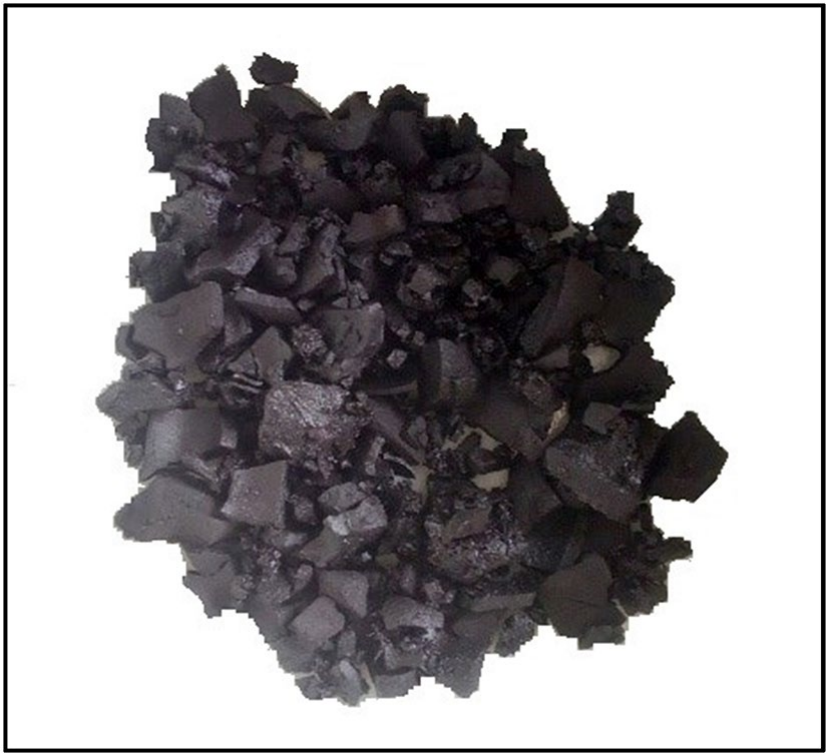
Physical appearance of HCP-AA.

Appearance of HCP-AA was amorphous and black in color that is shown in Fig. 1.

#### Physical properties of HCP-AA

Physical properties of HCP-AA are shown in Table 1, which shows that HCP-AA melts at temperature above 400 °C.

**Table 1:** Physical properties of HCP-AA.

Sr. No	Properties	Results
1	Melting Point	400 °C
2	Physical state	Amorphous
3	Color	Black

#### Chemical equation for synthesis of HCP-AA

**Figure 2:**
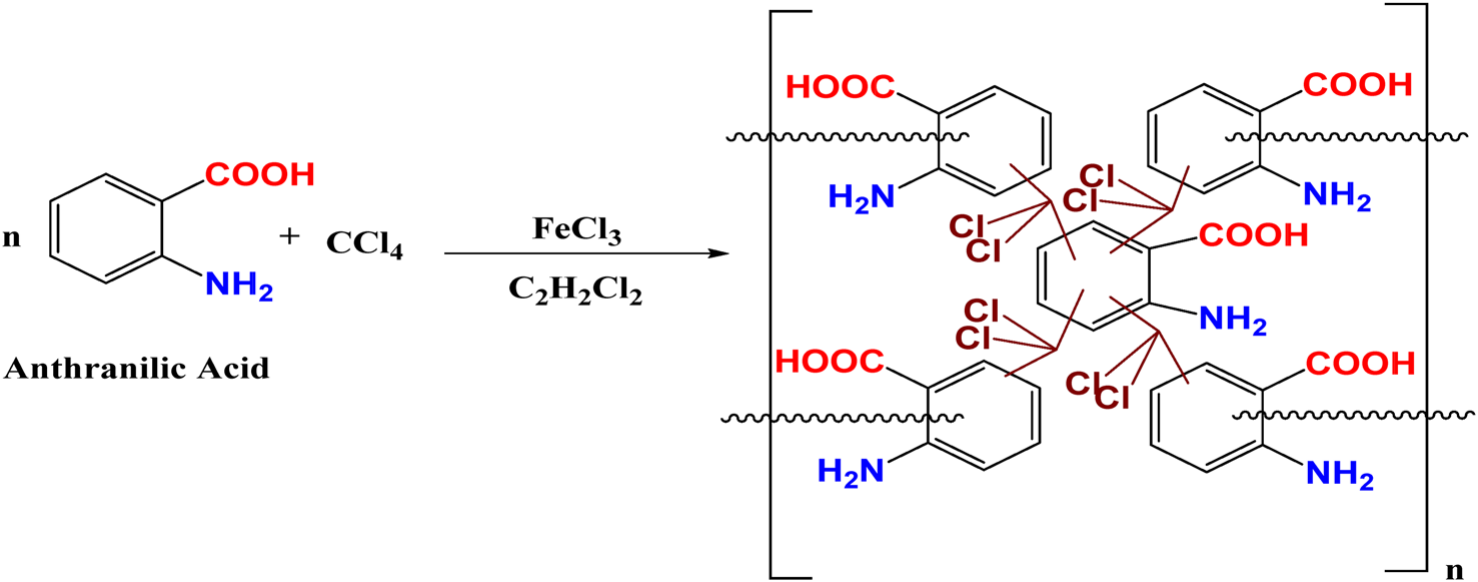
Chemical equation for synthesis of HCP-AA.

Chemical equation for the preparation of anthranilic acid based hyper cross-linked polymer is shown in Fig. 2.

#### Mechanism of synthesis of HCP-AA

**Figure 3:**
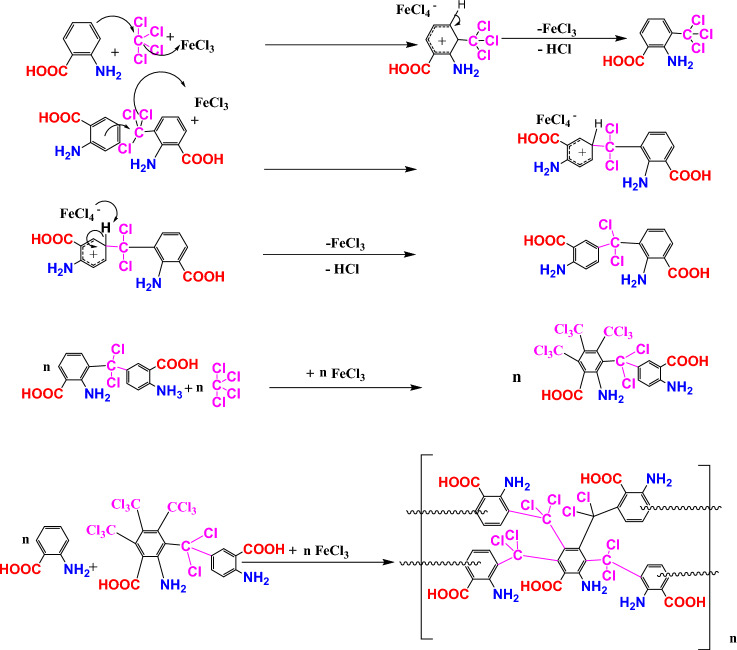
Possible reaction mechanism for synthesis of HCP-AA.

Figure 3 depicts the possible reaction mechanism for HCP-AA synthesis via Friedel Craft alkylation.

#### FT-IR results of HCP-AA

**Figure 4:**
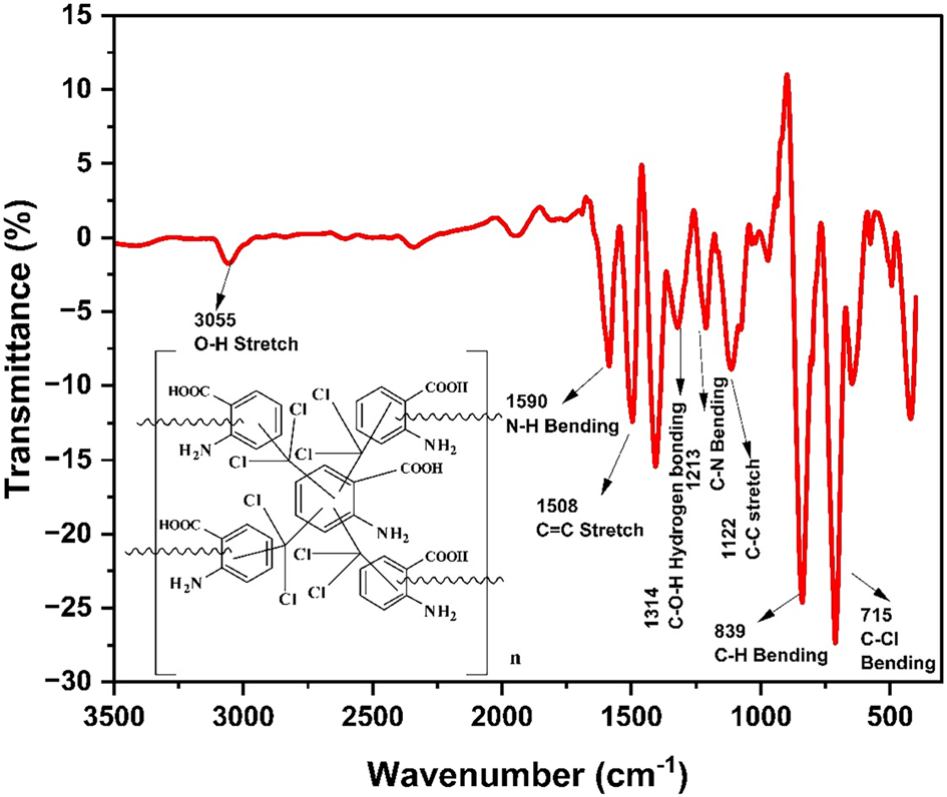
FTIR spectra of HCP-AA.

The FTIR spectrum of the HCP-AA is represented in Fig. 4. The peak, having medium intensity is shown at 1590 cm^−1^ that may indicates the presence of the N–H bond. The peak, having medium intensity which is observe at 1508 cm^−1^, indicates the presence of the C=C bond stretching in benzene ring. The hydroxyl group of carboxylic acid also involved in hydrogen bonding this is indicated by the presence of weak peak at 1314 cm^−1^ the presence of C–N bond is indicated by the weak peak at 1213 cm^−1^. Weak intensity peak at 1122 cm^−1^ indicates the presence of C–C bond of benzene ring. The most important and prominent peak at 715 cm^−1^ indicates the cross linking by C–Cl bond which may prove the synthesis of HCP-AA.

#### Energy dispersive X-ray spectroscopy

**Figure 5:**
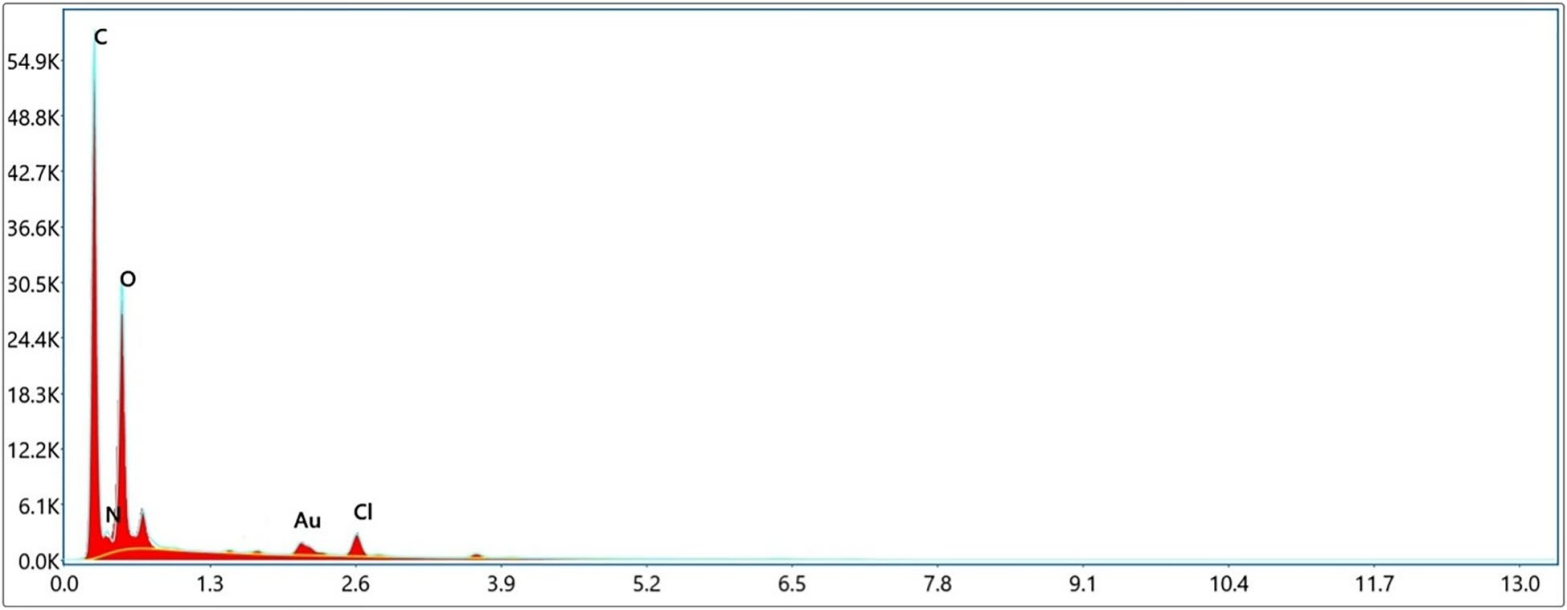
EDX graph showing composition of HCP-AA.

HCP-AA have carbon, nitrogen, oxygen and chlorine elements. The percentage of carbon was 65.84% that was the highest percentage of all other elements. Similarly, it had nitrogen of about 24.76, oxygen 7.87%, chlorine 1.32% as shown in Table 2 and Fig. 5. It also contains aurum 0.22% that might be come at the time of coating during SEM analysis.

**Table 2:** EDX results showing the percentage composition of HCP-AA.

Element	Weight%	Atomic%	Error%
Carbon	59.36	65.84	4.92
Nitrogen	26.03	24.76	13.23
Oxygen	9.45	7.87	16.45
Chlorine	2.24	1.32	20.34
Aurum	2.76	0.22	11.06

#### SEM and TEM results of HCP-AA

**Figure 6:**
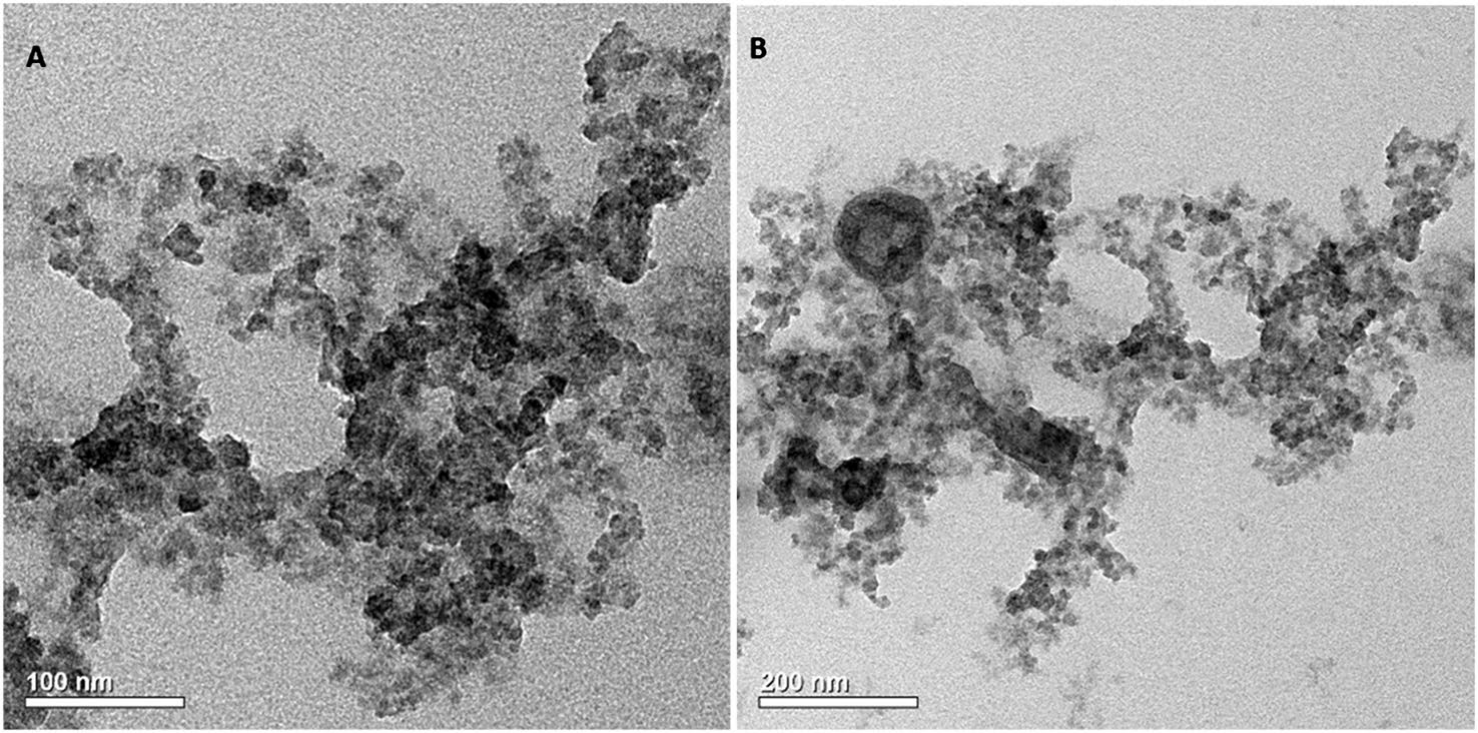
TEM Images of HCP-AA.

**Figure 7:**
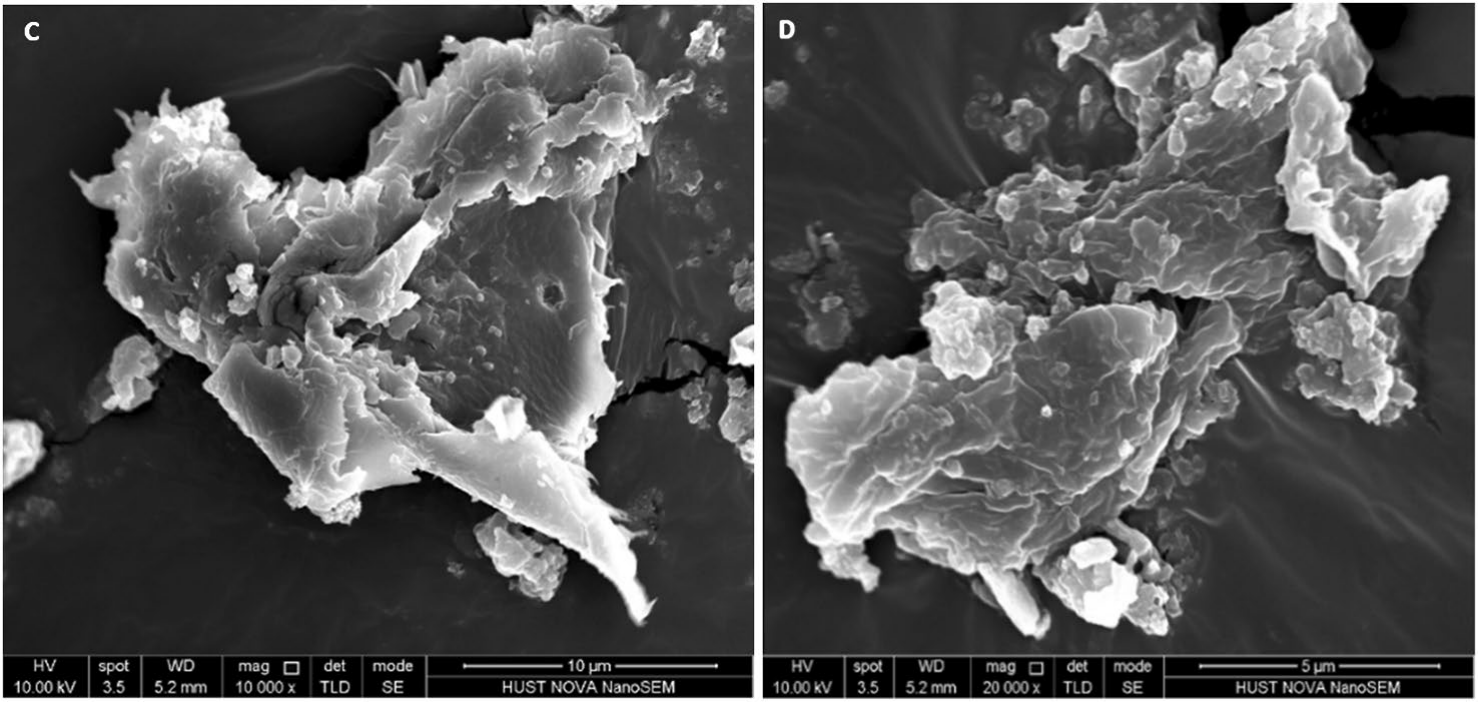
SEM images of HCP-AA.

The TEM and SEM analysis (Figs. 6 and 7) suggests that HCP-AA had porous structure and due to presence of abundant pores, it had excellent surface area. BET surface area of HCP-AA is 615 m^2^ g^−1^. Both SEM and TEM results prove that these materials are very suitable for uptake of Cr^3+^ and CO_2_ due to their porous structure.

#### XRD analysis

**Figure 8:**
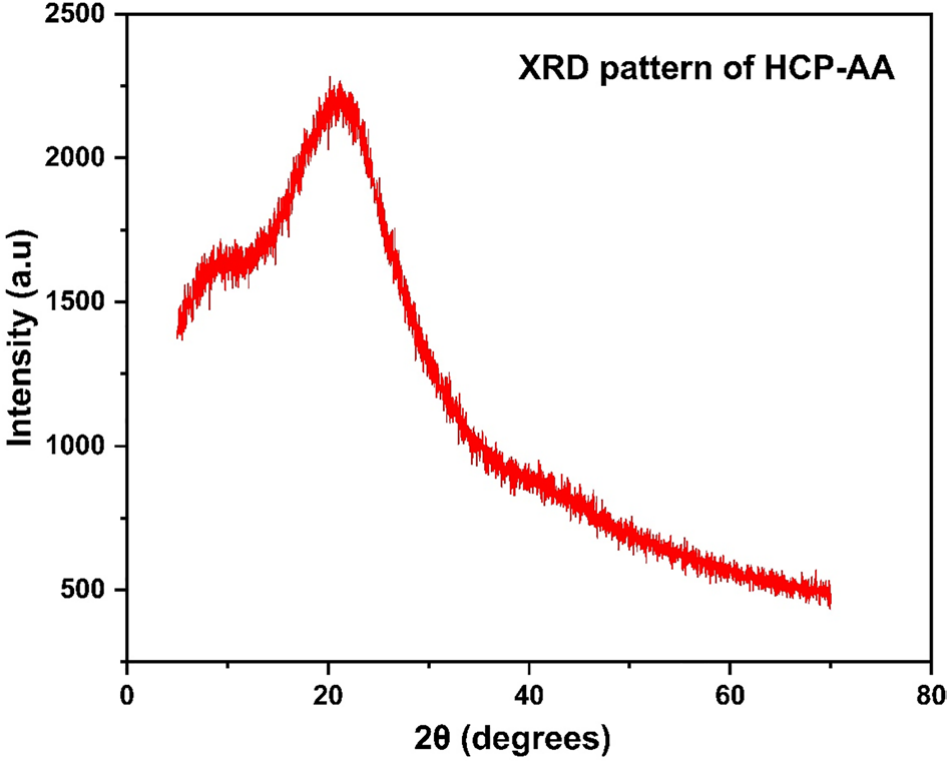
XRD graph of HCP-AA.

From XRD pattern of HCP-AA (Fig. 8), we conclude that there is no sharp peak at 2θ and it has some noisy pattern, which suggests that it is amorphous in nature.

#### Pore size distribution

**Figure 9:**
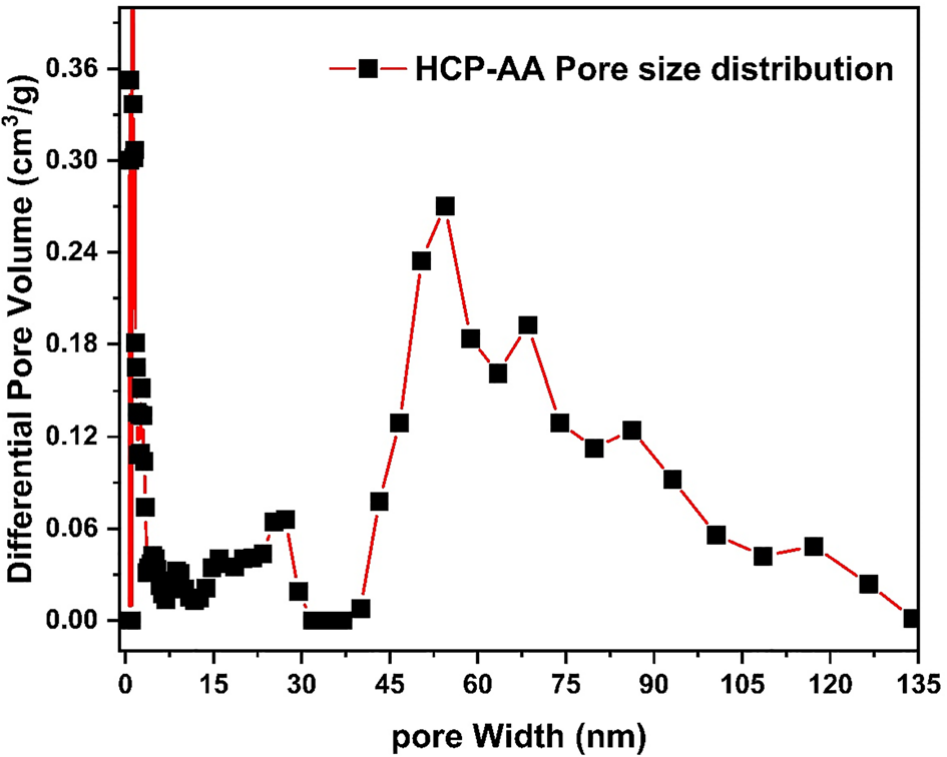
Pore size distribution of HCP-AA.

Pore size distribution of HCP-AA was examined. The results, extracted from Fig. 9, suggests that the pore width from ranges from 0.1 to 135 nm. It was measured by sorption analysis using nitrogen gas.

#### N_2_ adsorption–desorption isotherms

**Figure 10:**
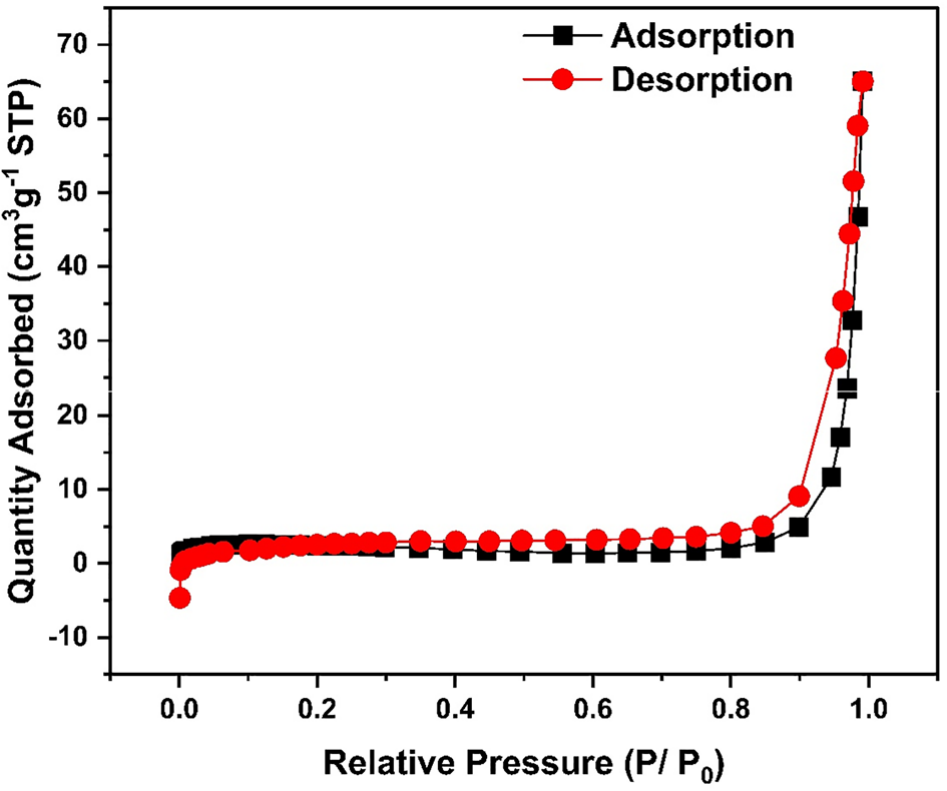
N_2_ adsorption—desorption isotherms of HCP-AA.

The N_2_ adsorption/ desorption isotherms of HCP-AA revealed the instantaneous uptake of N_2_ gas at low pressure which suggests that abundant number of micropores are present while the gas uptake at moderate pressure is attributed the presence of mesopores. Gas uptake at high pressure might be because of macropores as indicated in Fig. 10.

#### Thermogravimetric analysis

**Figure 11:**
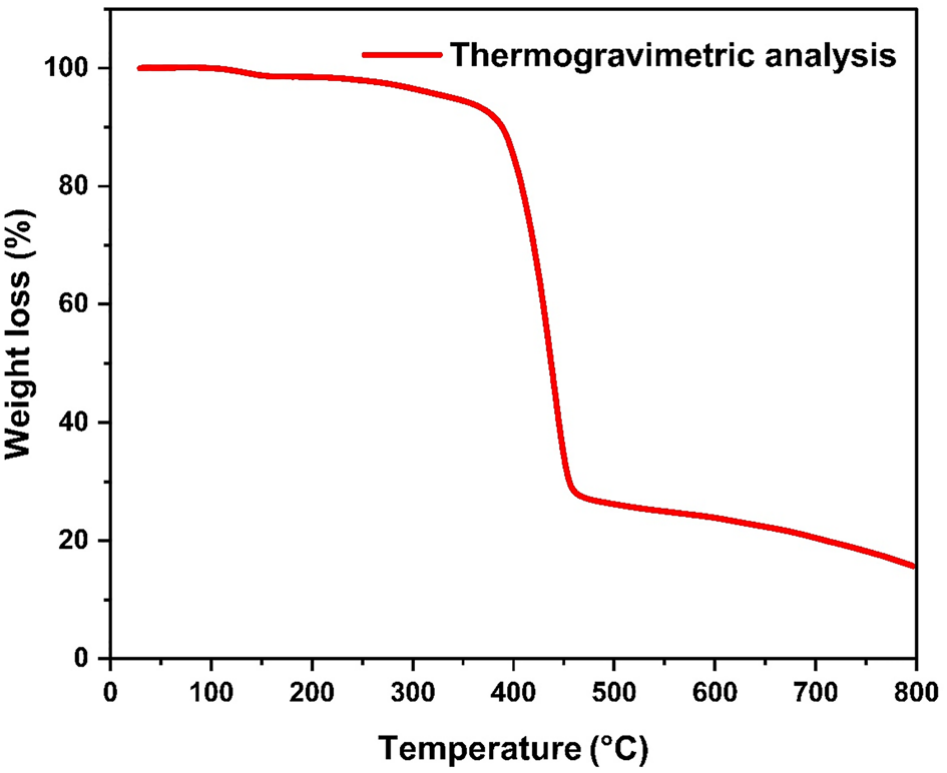
Thermogravimetric analysis of HCP-AA.

To further study the structural robustness and stability of HCP-AA, TGA was applied under inert nitrogen (N_2_) gas atmosphere where temperature ranges of 20–800 °C as shown in Fig. 11. It signified that the HCP-AA was stable up to 400 °C due to highly cross-linked structure and abundance of nitrogen groups. The small mass loss under 100 °C could be the result of moisture and trapped solvent.

#### UV–Vis spectroscopy

**Figure 12:**
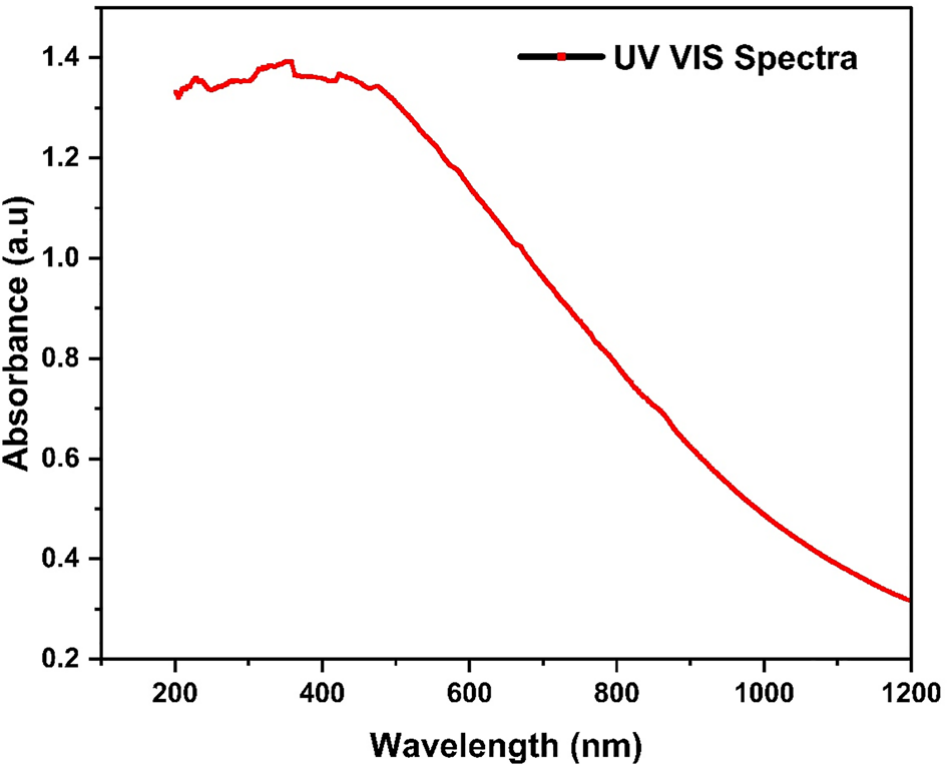
UV–Vis graph of HCP-AA.

All hyper cross-linked polymers showed absorption in both ultraviolet and visible region. HCP-AA also absorbed light in UV and VIS region and it showed lambda max at 428 nm with absorbance 1.389, which is shown in Fig. 12.

#### Application of HCP-AA in removal of heavy metals

HCPs of different aromatic compounds are excellent adsorbents having remarkable properties for the uptake of an organic pollutant, dyes and the heavy metals due to their low cost, easy synthesis and greater surface area with large number of pores. A standard solution of 1000 mg L^−1^ of chromium metal was prepared by mixing 1.631 g of CrCl_3_ to 1 L of distilled water. Standard solution of five different concentrations (10–50 mg L^−1^) was prepared with a gap of 10 mg L^−1^, respectively. The solutions of lower concentrations are difficult to analyze using AAS and the results are not reliable^59^. The synthesized HCP-AA is grinded well and thoroughly washed with ethanol followed by water until pH 7.0 was obtained. This is done to make sure the removal of all possible impurities. After this 0.8 g of purified HCP-AA was added to each prepared standard solution. These conical flasks were placed on the rotary shaker and stir at 160 rpm at 298 K for 12 min. The solution was filtered and 230ATS atomic absorption spectrophotometer (AAS) was applied to determine the concentration of Cr^3+^ from sample water. The AAS is a modern technique that is used for measurement of heavy metals in samples like the water. The percentage adsorption of HCP-AA for Cr^3+^ was 93% at optimum conditions. To perform adsorption experiment on real water sample, sample was collected from Royal leather industries Limited Lahore, Pakistan. The percentage adsorption of HCP-AA for Cr^3+^ in real wastewater sample is 88%. Decrease in percentage removal is caused by interfering ions present in real water sample. The percentage removal was calculated by using Eq. (1).

1 $${\text{Percentage removal of metal}} = \frac{{C_{o} - C_{e} }}{{C_{o} }} \times 100$$

#### FTIR analysis of HCP-AA before and after adsorption

**Figure 13:**
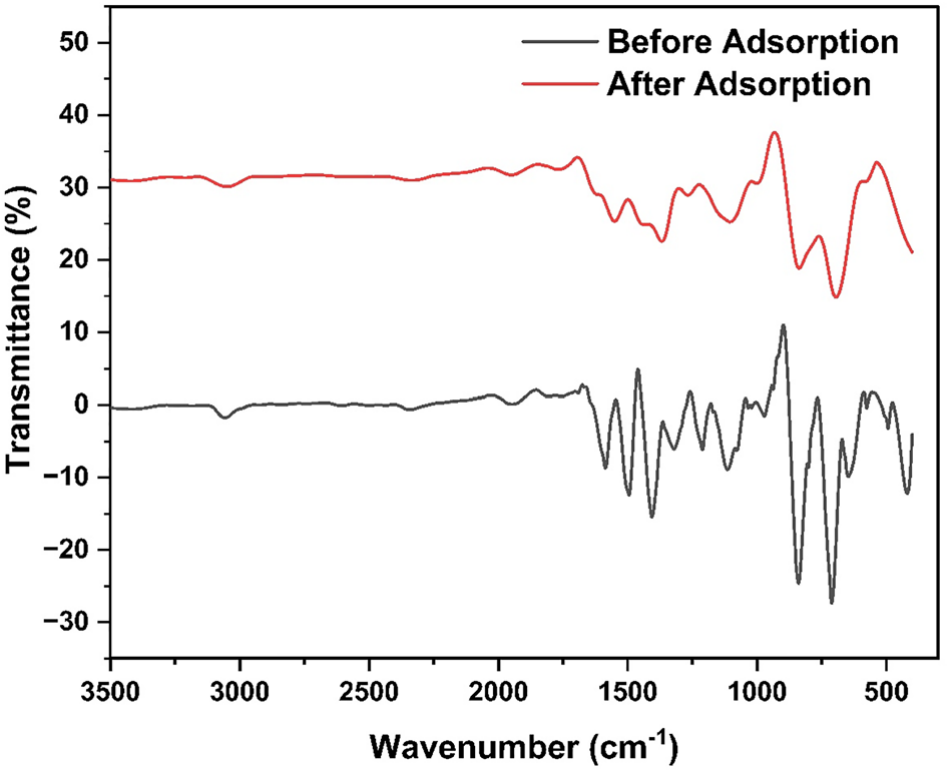
FTIR spectra of HCP-AA before and after an adsorption.

Figure 13 shows the FTIR spectra of HCP-AA before and after an adsorption of Cr^3+^ metal ions. The –OH group stretching peak appeared at 3055 cm^−1^ before adsorption, while after adsorption it is depressed and shifted to lower wavenumber (3045 cm^−1^), which may indicates the chemical adsorption of Cr^3+^ metal ions by –OH group of HCP-AA. This peak is not completely disappear after adsorption that may indicates that the HCP-AA is reusable for adsorption. The other peaks such as C–Cl bending 715 cm^−1^, C–H bending 839 cm^−1^ peaks merged, broaden and shifted to 687 cm^−1^ as chlorine may utilized its loan pair to adsorb Cr^3+^. While C–C stretching, C–N bending and C–O–H hydrogen bonding peaks were appeared in HCP-AA at 1122 cm^−1^, 1213 cm^−1^ and 1314 cm^−1^ respectively, these functionalities peaks also broaden, merged together and centered at 1128 cm^−1^ to justify their role in adsorption. Similarly peak 1508 cm^−1^ belonged to C=C stretching and N–H bending at 1590 cm^−1^ were depressed and broaden at position 1560 cm^−1^ to elaborate the fact that N nitrogen loan pair and pi-electronic cloud of C=C played a vital role in adsorption phenomenon. Some small sharp peaks appeared between the 450–550 cm^−1^ were merged to form a single wide peak at point 542 cm^−1^, which may indicates Cr and O interaction after the adsorption.

#### Mechanism of heavy metal adsorption

**Figure 14:**
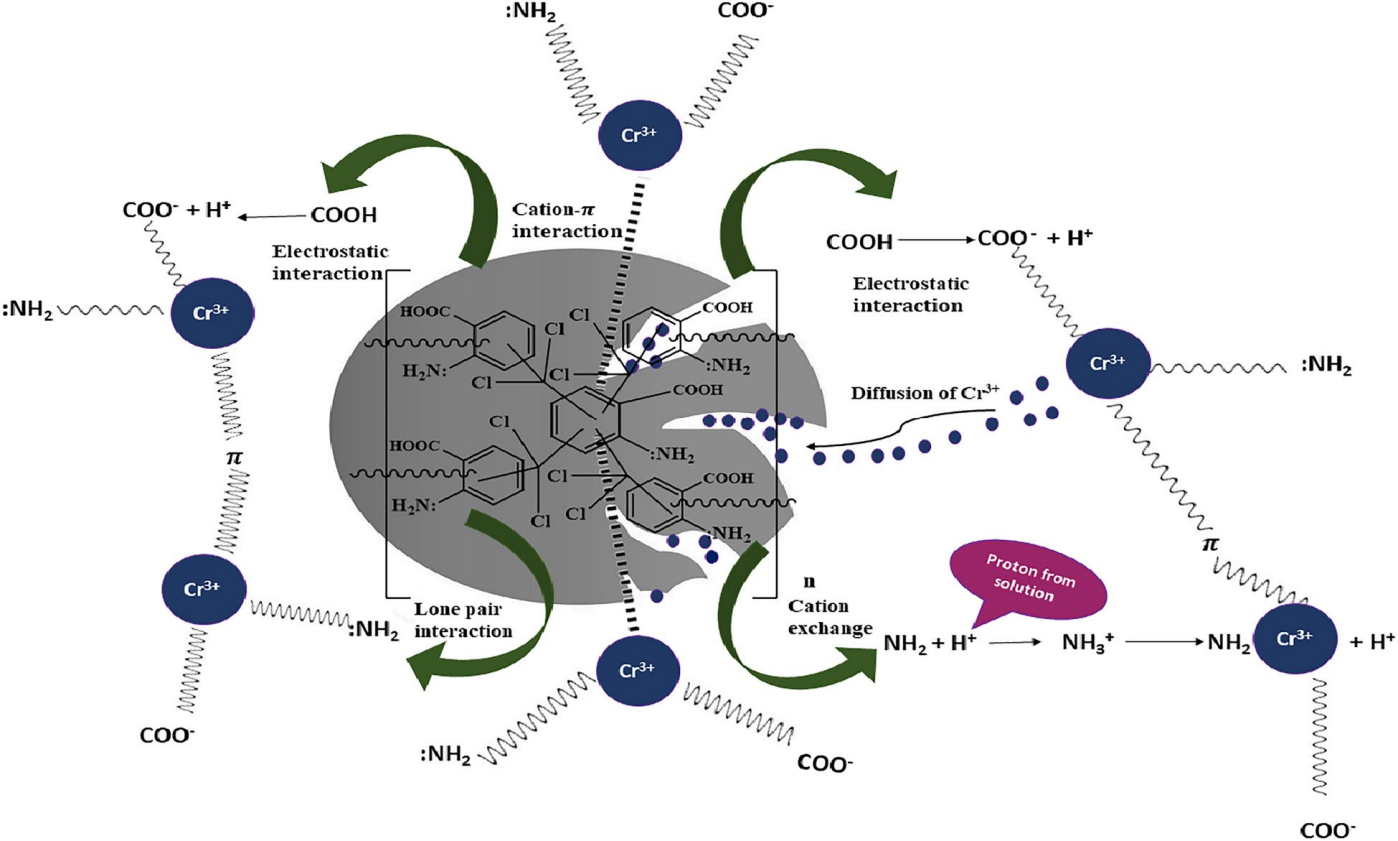
Mechanism for an adsorption of Cr^3+^ on surface of HCP-AA.

Nitrogen containing materials are attractive for the heavy metal ions uptake due to electron rich nature and there will be bond formation between electron deficient and electron rich centers (Lewis acid–base concept). Pi-electronic cloud of benzene ring also form interactions with positively charged heavy metals due to cation-$\pi$ interaction. Carboxyl group of HCP-AA after ionization form interactions with positively charged heavy metal ions. HCP-AA has porous surface so diffusion of heavy metals also takes place and it helps in adsorption of heavy metals. Reasonable surface area, narrow pore size distribution, hierarchical pore structure, accompanied by Lewis basic sites in HCP-AA are few of the desirable features for the study of heavy metal uptake where sorption isotherms measured at 273 K and 1 bar. The detailed mechanism of Cr^3+^ adsorption on the surface of HCP-AA is given in Fig. 14.

#### Effect of particle size of HCP-AA on heavy metal adsorption

The HCP-AA was transformed into powdered form using a pestle mortar and subsequently separated through different mesh size screens to obtain distinct particle sizes: 0.5 mm and 1 mm. Initial experiments revealed that the HCP-AA particles with a size of 0.5 mm exhibited superior adsorption capabilities compared to those with a 1 mm size. This can be attributed to the large surface area of the 0.5 mm particles. Given these findings, the 0.5 mm particle size was selected for subsequent experiments.

#### Point of zero charge (PZC)

**Figure 15:**
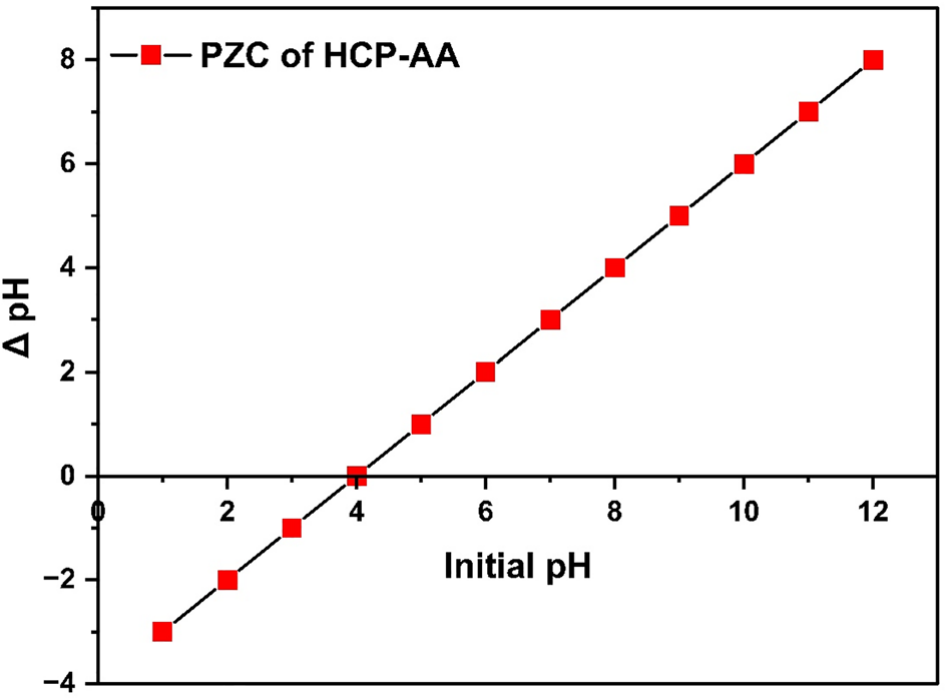
PZC of HCP-AA.

The point at which surface of adsorbent has zero net charge, because the positive and negative charges are equal in numbers, this phenomenon is known as PZC. The aim of this PZC study was to investigate the pH at which the adsorbent surface exhibited an equal quantity of opposing ions and how adsorbent efficiency could be increased with the change of pH. A graph is produced between pHi and ∆pH to measure PZC^60^. Figure 15, displays the value of PZC, which was obtained from a line intersecting the x-coordinate. Trials for calculating PZC by using the salt addition technique revealed that at pH 4.0, the HCP-AA had a PZC.

#### Effect of wastewater pH

**Figure 16:**
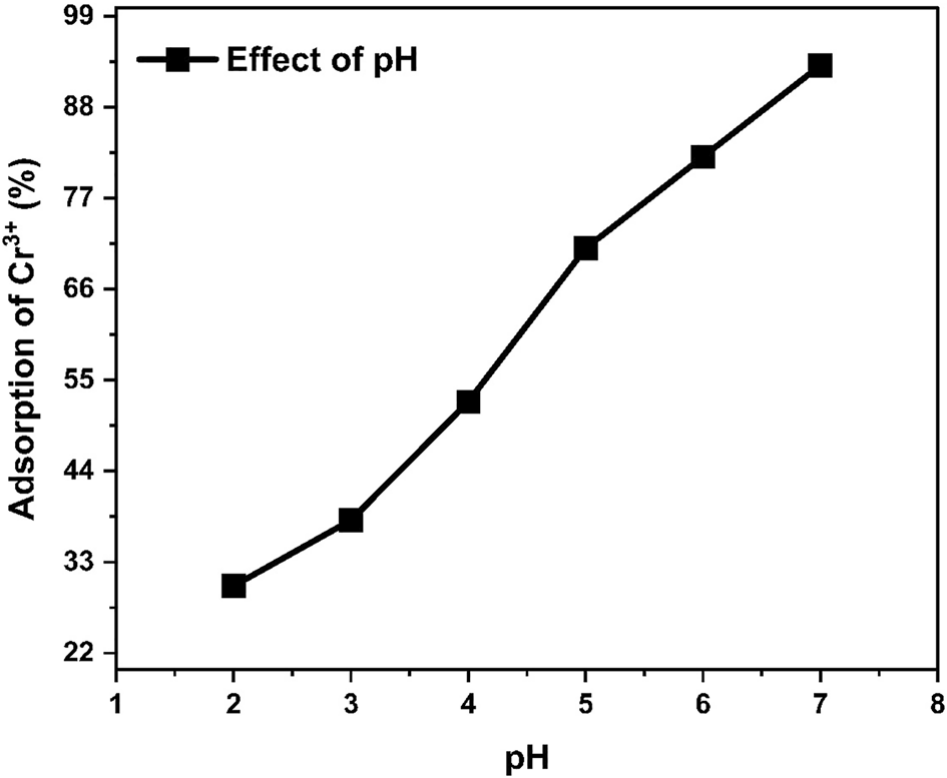
Effect of pH on adsorption.

Results revealed that, pH of wastewater also affected the adsorption by HCP. In the experiment, the pH changed from acidic to basic (2–7) which showed that adsorption was not uniform throughout the pH changes. There will be a chance of precipitation of chromium ions at basic pH that is why the pH (2–7) was maintained for experiments to get reliable results^61^. The highest Cr^3+^ adsorption took place at pH 7. Adsorption increased as the pH increased from point of zero charge, because at high pH, negative charge on adsorbent become dominant that caused strong electrostatic interaction between negatively charged surface and positive metal ion. Therefore, we conclude that optimum pH for adsorption by HCP takes place at pH 7 as revealed in Fig. 16.

#### Effect of adsorbent dose on adsorption

**Figure 17:**
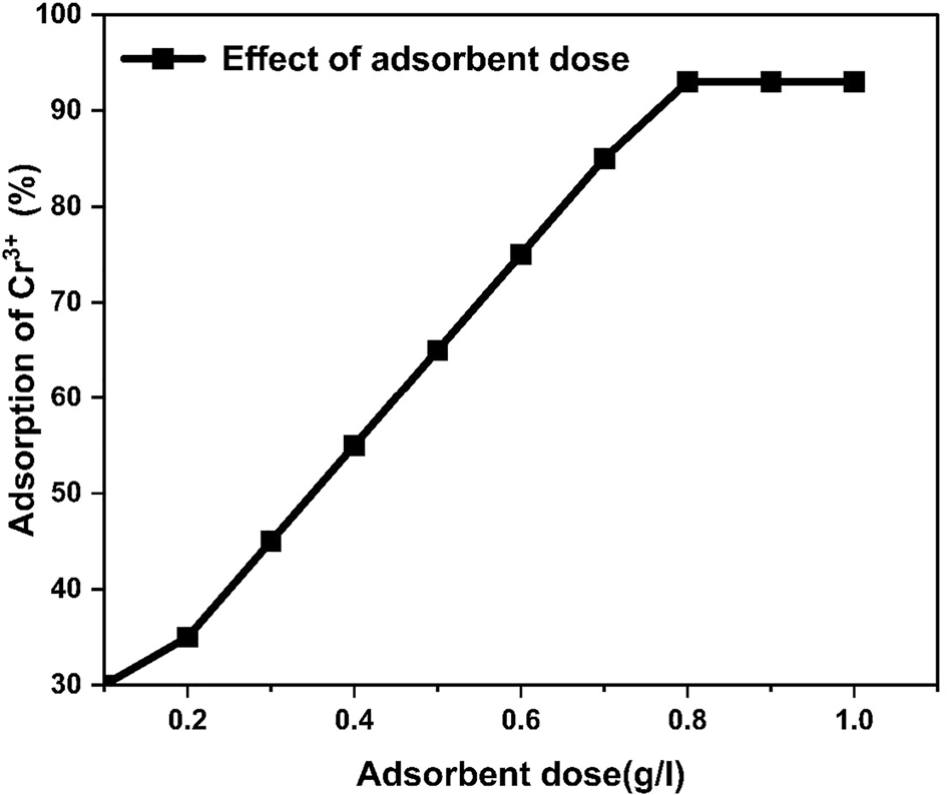
Effect of quantity of HCP-AA on adsorption.

Various quantities of HCP-AA, from 0.1 to 1.0 g, were tried and put into different flasks, containing 50 ml of the sample solution. Initially, the percentage removal of Cr^3+^ from water exhibited an upward trend as the quantity of adsorbent is raised (up to 0.8 g). The maximum Cr^3+^ removal was 93% at 0.8 g after this it became constant. This phenomenon may be due to the increased accessibility of active sites and a greater surface area when high dose of adsorbent was employed. A minor increase in removal efficiency after 0.8 g was because of equilibrium between the adsorbent HCP-AA and heavy metals. Therefore, we conclude that optimum quantity of adsorbent HCP-AA is 0.8 g, which is shown in Fig. 17.

#### Effect of contact time

**Figure 18:**
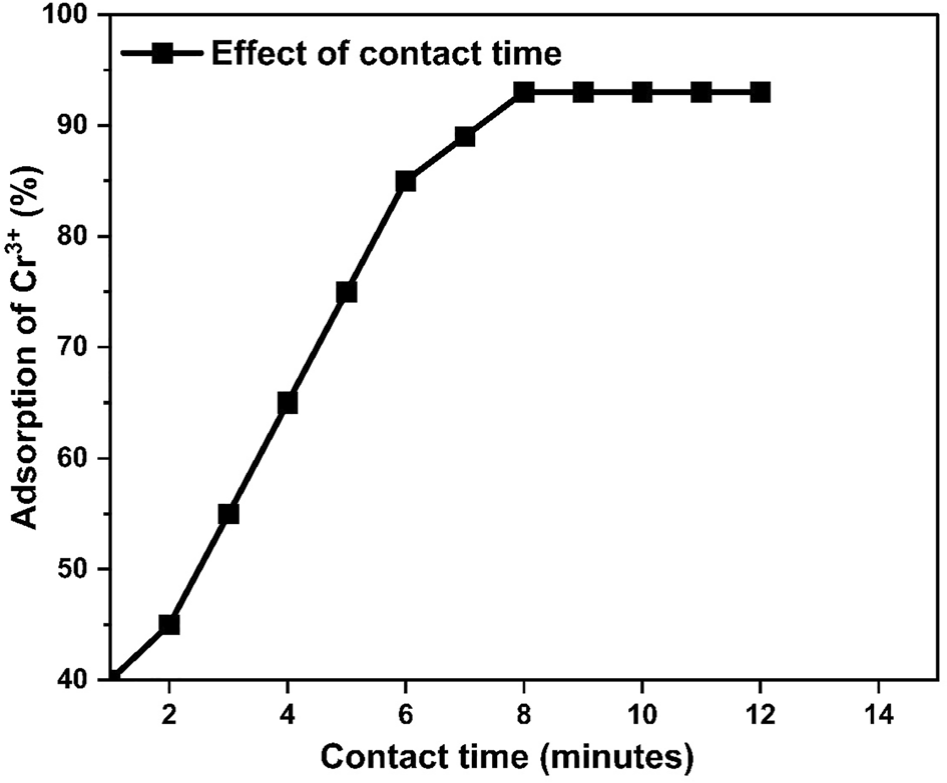
Effect of contact time on adsorption.

This study was done by differing the time of contact for adsorption from 1 to 12 min while keeping all other factors constant. At first, the removal percentage was increase at rapid rate in first 6 min due to the abundance of available empty spaces. After that, the rate of adsorption slowed down because lesser sites were available for adsorption of Cr^3+^. The maximum an adsorption took place at the time of 8 min, which was 93% and then became constant so, we conclude that optimum contact time for adsorption is 8 min as depicted in Fig. 18.

#### Effect of temperature

**Figure 19:**
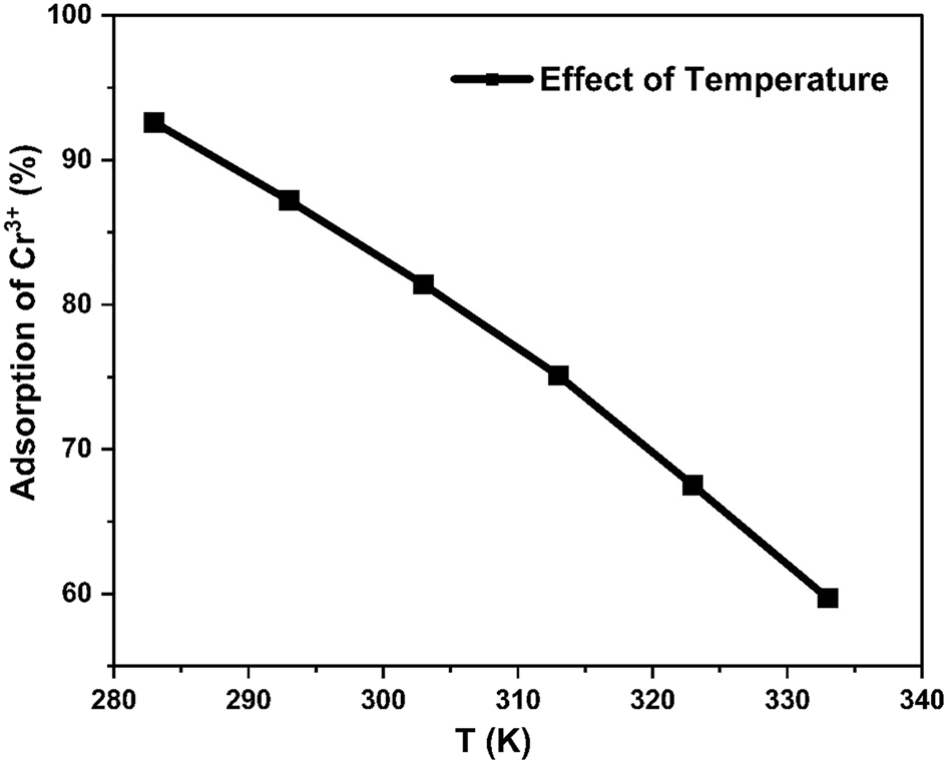
Effect of temperature on adsorption.

The influence of temperature on uptake of Cr^3+^ onto surface of HCP-AA was examined in a range of temperatures, including 0–60 °C, while maintaining constant conditions of initial concentration 0.8 g/L, 12-min contact time, and a pH of 7.0. The findings revealed a proportional decrease in adsorption percentage with rising temperature, indicating an inverse correlation between temperature and the percentage removal of the heavy metals. Maximum adsorption occurred at lowest temperatures, resulting in a percentage removal variation from 92.6 to 59.7% within the temperature range of 283–333 K. These results are visually presented in Fig. 19. The enhanced adsorption process at minimum temperatures attributed to an augmentation in binding sites on the surface of HCP-AA.

### Adsorption isotherms

#### Langmuir isotherm

The Langmuir isotherm parameters are shown in Table 3. The parameter RL revealed an adsorption is favorable for these metals ions. These parameters were calculated by using the following relation;

2 $${\text{RL}} = \frac{1}{{1 + b \cdot q_{m} }}$$

**Table 3:** Langmuir Isotherm data for chromium metal.

No. of experiment	C_o_ (mg/L)	C_e_ (mg/g)	1/C_e_	Q_e_ (mg/g)	1/Q_e_
1	5	2.38	0.42017	3.486429	0.286826
2	10	8.7	0.11494	6.832143	0.146367
3	20	13.25	0.07547	13.8125	0.072398
4	50	52.84	0.01893	33.82714	0.029562
5	100	59.68	0.01676	69.29714	0.014431
6	150	63.27	0.01581	104.8832	0.009534

**Figure 20:**
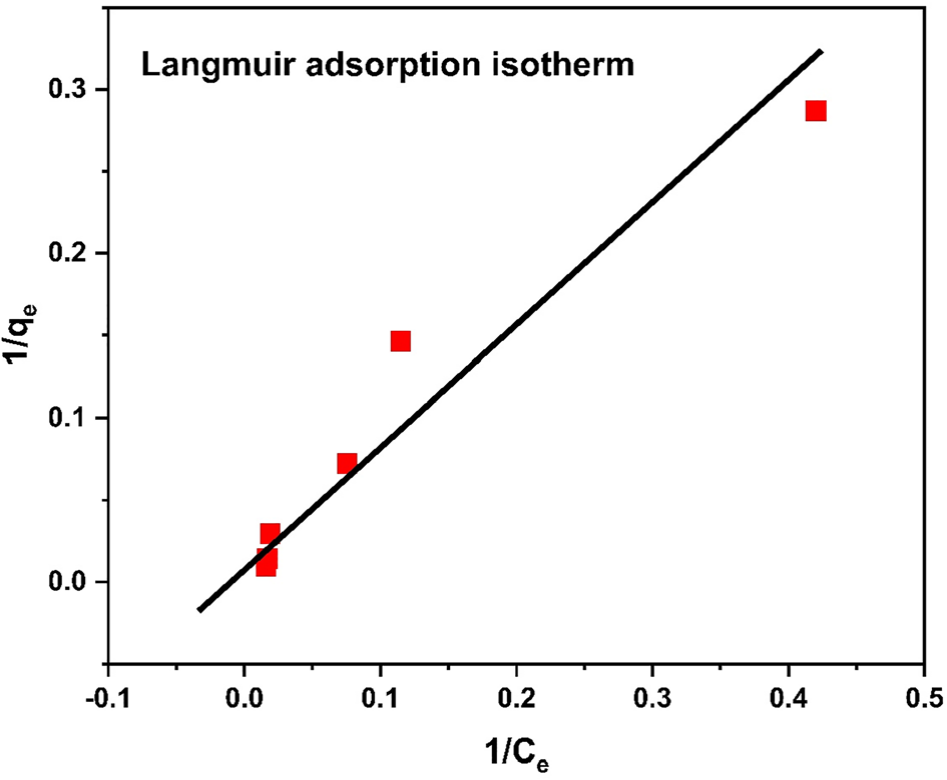
Langmuir isotherm for chromium metal.

The RL value is between 0 and 1 which confirms the successful uptake of Cr^3+^. The Langmuir adsorption isotherm that is shown in Fig. 20 is excellent model to study monolayer adsorption and is mostly applied to find adsorption parameters for studies.

The linear form of the Langmuir equation is shown in Eq. (3).

3 $$\frac{{P_{e} }}{{q_{e} }} = \frac{1}{{q_{m \cdot b} }} = \frac{{P_{e} }}{{q_{m} }}$$

The resulted data were well fitted by using Langmuir isotherm model^62^.

#### Freundlich isotherm

From the data obtained from Freundlich adsorption isotherm which is reported in Table 4 shows that 1/n = 0.731 while n = 1.37 shows an adsorption of Cr^3+^ is favorable.

**Table 4:** Experimental data of Freundlich isotherm.

No. of experiment	C_o_ (mg/L)	C_e_ (mg/g)	Log C_e_	Q_e_ (mg/g)	Log Q_e_
1	5	2.38	0.3765	3.486429	0.5424
2	10	8.7	0.9395	6.832143	0.8346
3	20	13.25	1.1222	13.8125	1.1403
4	50	52.84	1.7229	33.82714	1.5293
5	100	59.68	1.7758	69.29714	1.8407
6	150	63.27	1.8012	104.8832	2.0208

Mathematical form of the Freundlich isotherm equation is given below;

4 $$Q_{e} = K_{f } C_{e}^{\frac{1}{n}}$$

The Freundlich isotherm's linear form is given here;

5 $$\log Q_{e} = \log K_{f } + \frac{1}{n}\log C_{e}$$

**Figure 21:**
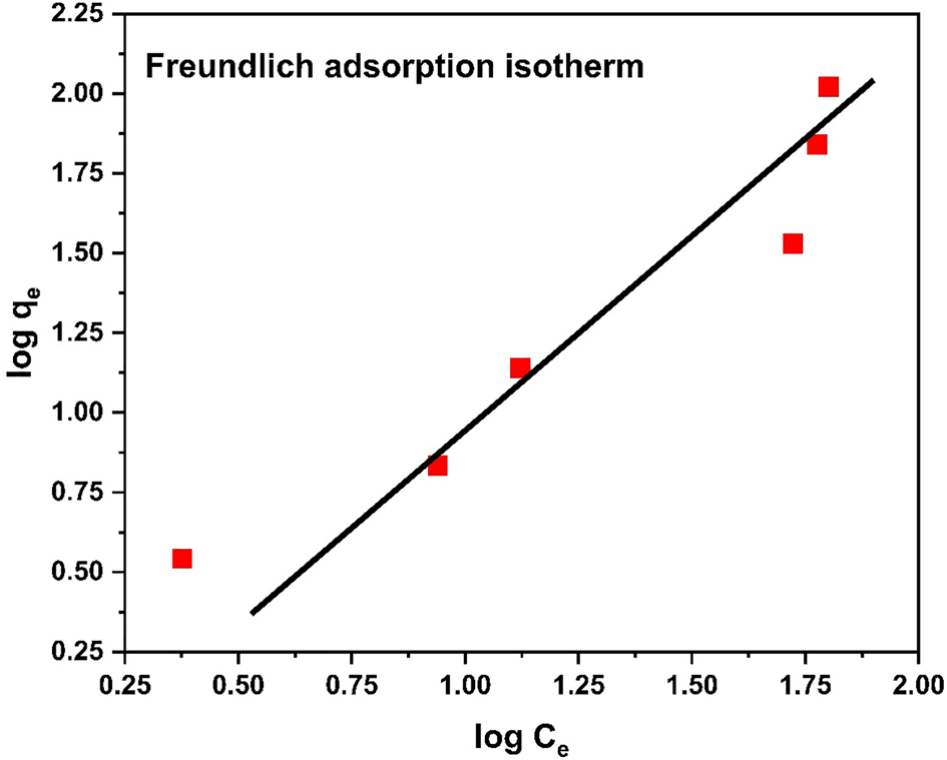
Freundlich Isotherm.

The Freundlich adsorption isotherm graph is shown in Fig. 21.

#### Temkin isotherm

Temkin isotherm experimental analysis is shown in the Table 5. The R^2^ value of Temkin isotherm is 0.6654, which shows that this adsorption is favorable.

**Table 5:** Experimental data of Temkin isotherm.

No. of Experiment	C_o_ (mg/L)	C_e_ (mg/g)	Log C_e_	Q_e_ (mg/g)
1	5	2.38	0.3765	3.486429
2	10	8.7	0.9395	6.832143
3	20	13.25	1.1222	13.8125
4	50	52.84	1.7229	33.82714
5	100	59.68	1.7758	69.29714
6	150	63.27	1.8012	104.8832

Mathematical form of Temkin isotherm model is following

6 $${\text{Q}}_{{\text{e}}} = {\text{ RT}}/{\text{b }} \times {\text{ ln }}\left( {{\text{A}}_{{\text{r}}} {\text{C}}_{{{\text{aq}}}} } \right)$$

Linear form of the Temkin model

7 $${\text{Q}}_{{\text{e}}} = {\text{ B}}_{{1}} {\text{logK}}_{{\text{t}}} + {\text{ B}}_{{1}} {\text{logC}}_{{\text{e}}}$$

**Figure 22:**
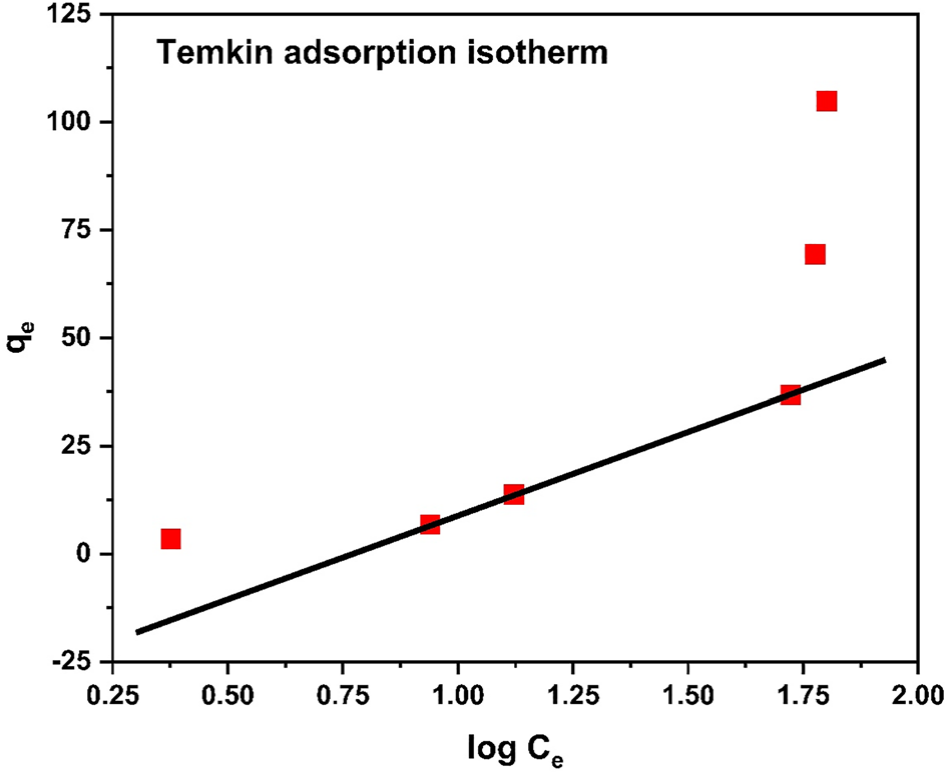
Temkin Isotherm.

The Temkin adsorption isotherm graph is shown in Fig. 22**.**

#### Dubinin Radushkevich isotherm

Employing the Dubinin–Radushkevich isotherm, the mechanism of adsorption is expressed through Gaussian energy distribution onto a heterogeneous surface of the adsorbent.

8 $${\text{q}}_{{\text{e}}} = \, \left( {{\text{q}}_{{\text{s}}} } \right){\text{exp }}( - {\text{K}}_{{{\text{ad}}}} \varepsilon^{{2}} )$$

9 $${\text{lnq}}_{{\text{e}}} = {\text{ln}}\left( {{\text{q}}_{{\text{s}}} } \right) - \left( {{\text{K}}_{{{\text{ad}}}} \varepsilon^{{2}} } \right)$$

where q_e_, q_s_, K_*ad*_, ε are

- q_e_ = amount of an adsorbate in the adsorbent at the equilibrium(mg/g);
- q_s_ = theoretical isotherm saturation capacity (mg/g);
- *K*_*ad*_ = Dubinin–Radushkevich isotherm constant (mol^2^/kJ^2^) and
- ε = Dubinin–Radushkevich isotherm constant^63^*.*

This model aids in differentiating between the physical and chemical adsorption of heavy metal ions to calculate the mean free energy E per molecule of adsorbate.

10 $$E = 1/\sqrt {2B_{{{\text{DR}}}} }$$

**Figure 23:**
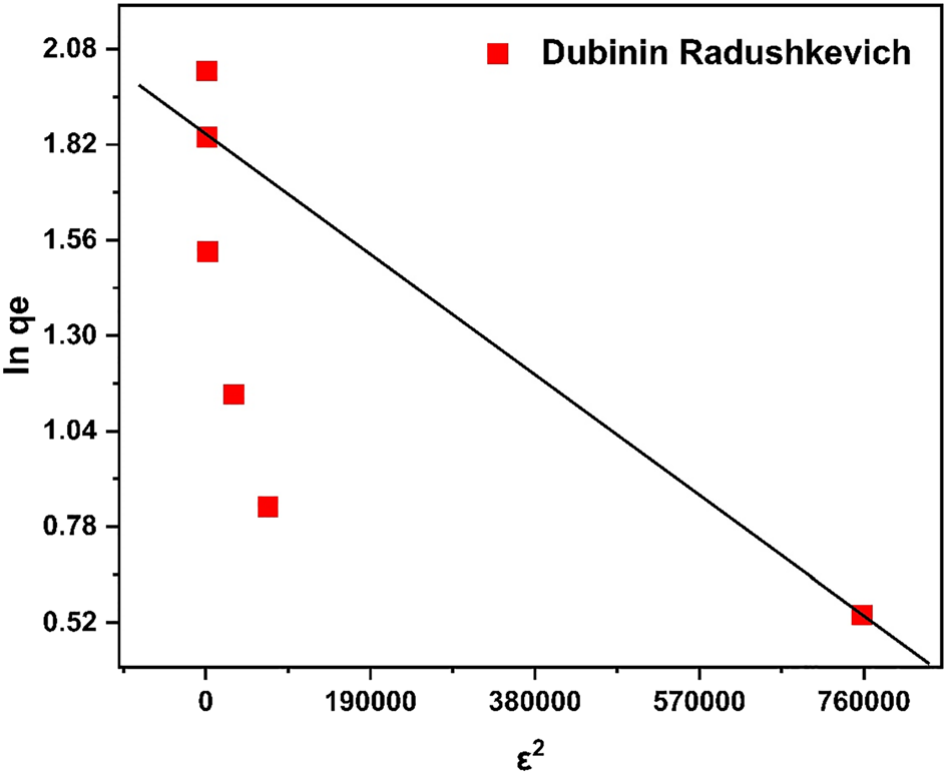
Dubinin Radushkevich Isotherm.

In the meantime, the value of ε can be determined as follows:

11 $$\varepsilon = {\text{ RTln}}\left[ {{1} + {1}/{\text{Ce}}} \right]$$

where Ce, T, and R stand for the adsorbate equilibrium concentration (mg/L), absolute temperature (K), and gas constant (8.314 J/mol K), respectively. The Dubinin-Radushkevich isotherm model is well known for the identification of its temperature-dependent feature, which is demonstrated when adsorption data at various temperatures are plotted as a function of the amount-adsorbed logarithm. lnq_e_ versus ε^2^ that is shown in Fig. 23, ε^2^ is the square of potential energy, all suitable data will lie on the same curve, named as the characteristic curve. The constants such as q_s_, and *K*_*ad*_ were calculated from the appropriate plot using equation No. 11^63^. From the linear plot of DRK model, q_s_ was determined to 55.3 mg/g, the mean free energy, E = 0.7 kJ/mol indicates a physiosorption process with the *R*^2^ = *0.5125.*

The comparison of isotherms parameters is illustrate in Table 6. The above data revealed that the adsorption can be best explained by Freundlich adsorption isotherm with R^2^ = 0.9273. Study of isotherms revealed multilayer adsorption and adsorption capacity increases with increase in concentration of chromium ions. However close R^2^-value of Langmuir to Freundlich indicated monolayer adsorption with q_max_ of 52.63 mg/g, which confirm heterogeneous nature of adsorbent surface.

**Table 6:** Comparison of adsorption isotherm parameters.

Adsorption isotherms	R^2^	Other parameters	
Langmuir	0.91	q_m_(mg/g)	69.67
		K_L_ (L/g)	0.013
Freundlich	0.9273	N	1.036
		1/n	0.965
		K_f_	1.183
Temkin	0.6654	B_t_	56.34
		K_t_	0.250

#### Kinetics study

Rate of adsorption was determined through kinetics study, which is discussed below, and it showed that an adsorption is well fitted with a pseudo second order model.

#### Pseudo first order reaction

**Figure 24:**
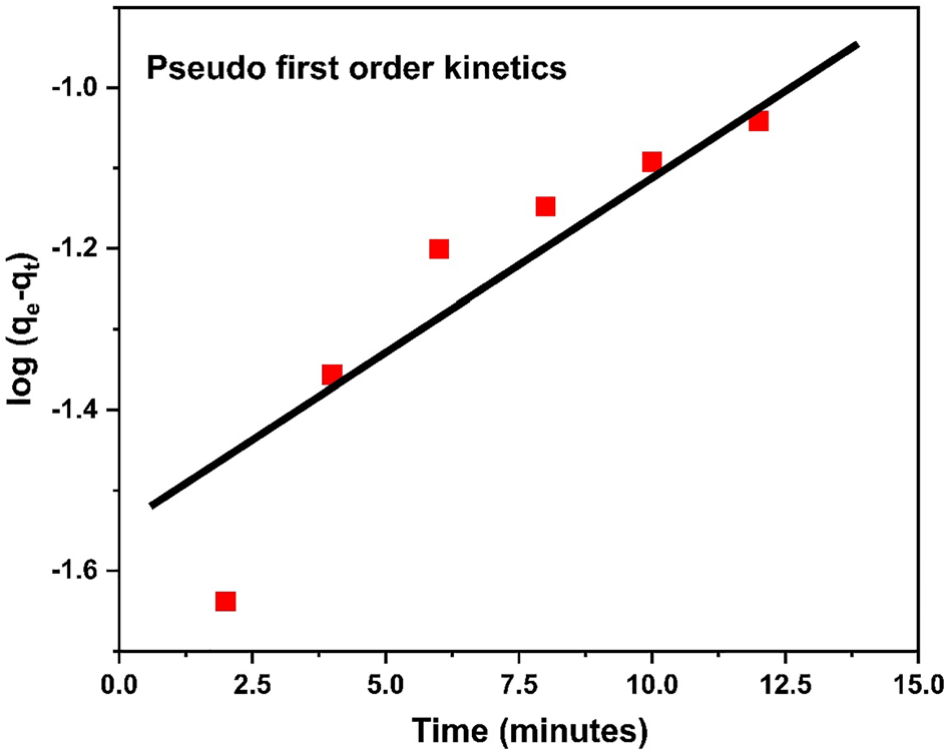
Pseudo first order kinetics graph.

Linear equation of pseudo first order reaction is as follow

12 $$\log \left( {Q_{e} {-} Q_{t} } \right) = \log Q_{e} {-} \left( {k_{1} /2.302 } \right) \times t$$

where Q_e_ is equilibrium concentration, Q_t_ is adsorption concentration at the time t and K is constant. Figure 24 describes the pseudo first kinetic model of adsorbent towards adsorption.

#### Pseudo second order reaction

Equation of the pseudo second order reaction is given below

13 $${\text{Q}}_{{\text{t}}} = {\text{ q}}_{{\text{e}}}^{2} {\text{K}}_{2} {\text{t}}/1 + {\text{q}}_{{\text{e}}} {\text{K}}_{2} {\text{t}}$$

**Figure 25:**
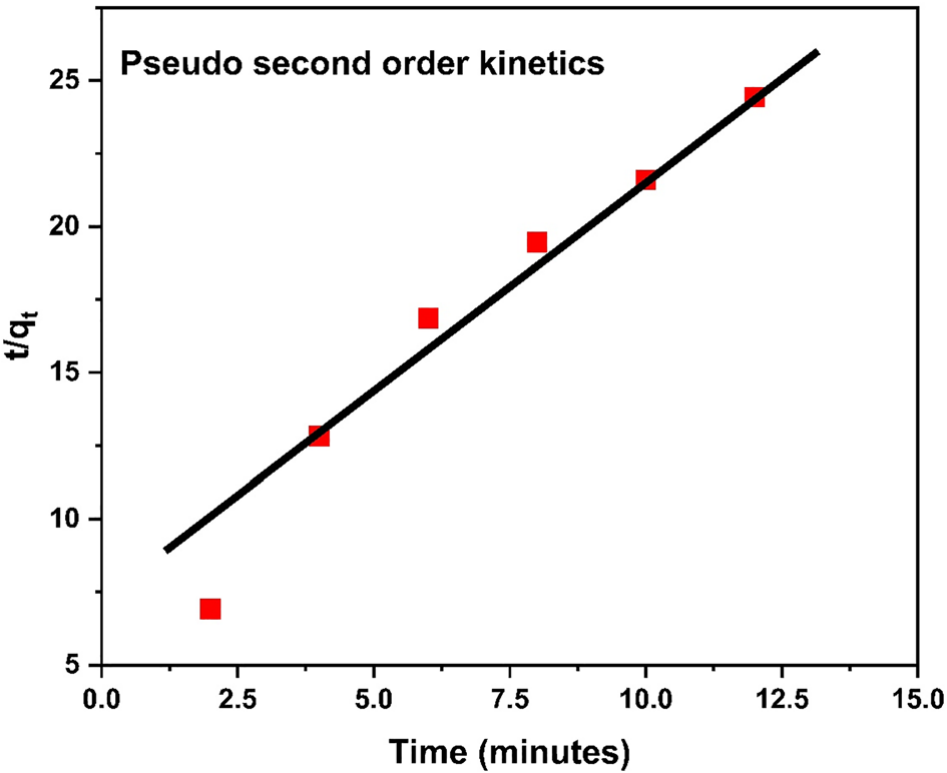
Pseudo second order kinetics graph.

Figure 25 describes a pseudo second kinetic model behavior of adsorbent towards the adsorption.

#### Intraparticle diffusion model

**Figure 26:**
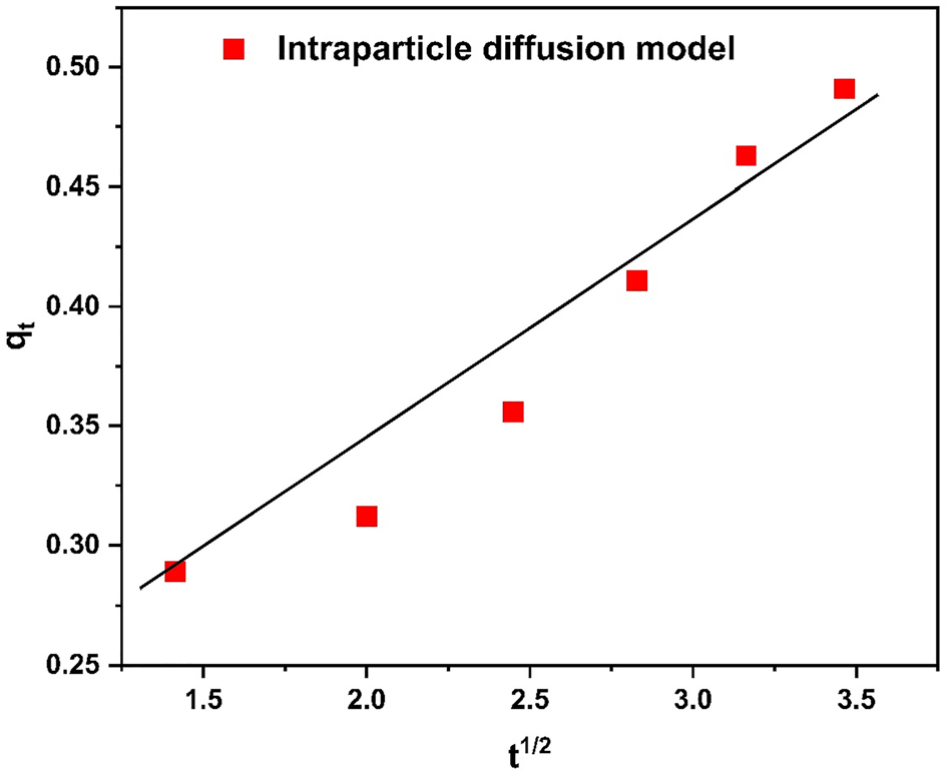
Intraparticle diffusion model.

Weber and Morris present an intraparticle diffusion model for finding out the adsorption process's diffusion mechanism and rate-controlling phase. This model's mathematical representation is as follows:

14 $${\text{qt }} = {\text{ Kidt}}^{0:5} + \, I$$

where I is the layer’s thickness, qt(mg/g) is the amount of adsorbate adsorbed at time “t” and kid is intra-particle diffusion constant. The plot produced by the data is linear, a value of regression coefficient, is 0.9582^64^. The value of Kdiff is 0.1052 and thickness of layer on surface of HCP-AA is 0.1186. The graph between t^1/2^ and q_t_ is shown in Fig. 26.

According to the comparative study it is observed that pseudo second order model is fit the best with R^2^ = 0.979 as compares to pseudo 1st order kinetics model. The second order kinetic model predicts chemical adsorption of Cr^3+^ on surface of polymer. The 1st order kinetic model's regression “R^2^” value “0.601” is lower than the 2nd order kinetic model’s R^2^ value 0.979. Additionally, q_e_ calculated (0.601 mg g^−1^) value from Eq. 8 and 9 is closer to q_e_ experimental value (0.589 mg g^−1^) as shown in Table 7. Therefore, second order kinetic model is the best to examine the kinetic constants of Cr^3+^ adsorption on surface of HCP-AA. Comparative data analysis is shown in Table 7.

**Table 7:** Comparison of kinetics graph.

Sr. No	Order of reaction	Intercept	R^2^	Q_e_ exp	Q_e_ cal	K
1	Pseudo first order	− 1.589	0.861	0.589	0.023	K_1_ (min^−1^) = 0.125
2	Pseudo second order	5.536	0.979	0.589	0.601	K_2_ (gmol^−1^ min^−1^) = 0.52

#### Thermodynamics study

The determination of heat changes in a system, or its state, can be described using various state functions, such as the Gibbs free energy (∆G), an entropy (∆S), and the enthalpy (∆H). These parameters offer insights into the mechanism of the adsorption, distinguishing between exothermic and endothermic reactions. The thermodynamic relationships can be expressed through the equation:

15 $$\ln K_{c} = \left( {\Delta S/R} \right) {-} \left( {\Delta H/RT} \right)$$

here

- ΔS represents entropy (indicating degree of randomness),
- ΔH denotes enthalpy
- T is a temperature in Kelvin
- K_c_ is an equilibrium constant

An equilibrium constant (K_c_) is defined as the ratio of a amount of heavy metal adsorbed on surface of HCP-AA at the equilibrium (C_a_) to equilibrium concentration of a heavy metal in the solution (C_e_), as given by:

16 $$K_{c} = C_{a} {/}C_{e}$$

In exothermic processes, the Gibbs free energy (Δ*G*^0^), a entropy (Δ*S*^0^), and the enthalpy (Δ*H*^0^) exhibit negative values, governed by the relationship:

17 $$\Delta G^{0} = \Delta H^{0} - T\Delta S^{0}$$

**Figure 27:**
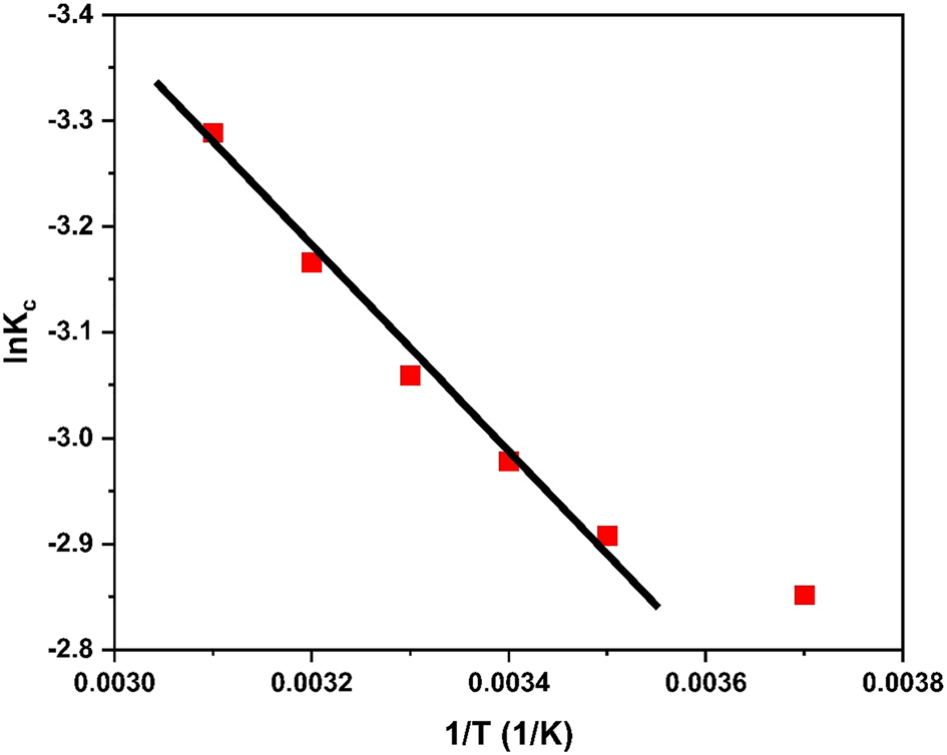
Graph of thermodynamics study.

Negative enthalpy and entropy value signify the exothermic nature of the reaction, suggesting an inverse relationship with temperature. As temperature increases, adsorption tends to decrease, and vice versa. The graph between ln *Kc* versus 1/*T* for the adsorption of chromium over HCP-AA is illustrated in Fig. 27. The value for adsorption enthalpy (Δ*H*^0^) was − 60.91 kJ/mol and adsorption entropy (Δ*S*^0^) was − 45.79 kJ/mol K which were calculated from plot. The resulting values for Gibbs free energy (Δ*G*^0^) are presented in Table 8. The Positive Δ*G*^0^ suggests the non-spontaneous nature of a process. A rise in the Δ*G*^0^ values with increase in temperature shows adsorption is unfavorable at the higher temperatures. A negative Δ*H*_0_ value implies that the adsorption of Cr^3+^ is exothermic, while the negative Δ*S*^0^ value shows a reduction in unpredictability at the contact between the adsorbent-adsorbate during the adsorption.

**Table 8:** Thermodynamics study of adsorption.

T (K)	1/T (1/K)	C_e_	K_c_	lnK_c_	ΔG (KJ/mol)	ΔS (J/mol k)
283	0.0037	0.76	0.05775	− 2.8516	6.5880	− 0.238
293	0.0035	1.28	0.05459	− 2.9079	7.0836	− 0.232
303	0.0034	1.86	0.05087	− 2.9784	7.5030	0.226
313	0.0033	2.49	0.04693	− 3.0591	7.9606	− 0.220
323	0.0032	3.25	0.04218	− 3.1658	8.5015	− 0.213
333	0.0031	4.03	0.03731	− 3.2884	9.1041	− 0.210

The adsorption capacity of HCP-AA in relation to other comparable adsorbents is displayed in Table 9. It shows 52.63 mg/g adsorption capacity of HCP-AA that is due to is high surface area, porous surface and active functional groups.

**Table 9:** Comparison of HCP-AA with other adsorbents for chromium removal.

Material	Q_max_ (mg/g)	References
SAM-HCPs	51	^65^
FIR-54	53.2	^66^
PEI/ECs	36.8	^67^
HCPs-N	44.5	^68^
PVIm-6-SCD	236.8	^69^
MC-N	14.8	^70^
HCP-AA	52.63	This work

#### Application of HCP-AA for CO_2_ uptake

**Figure 28:**
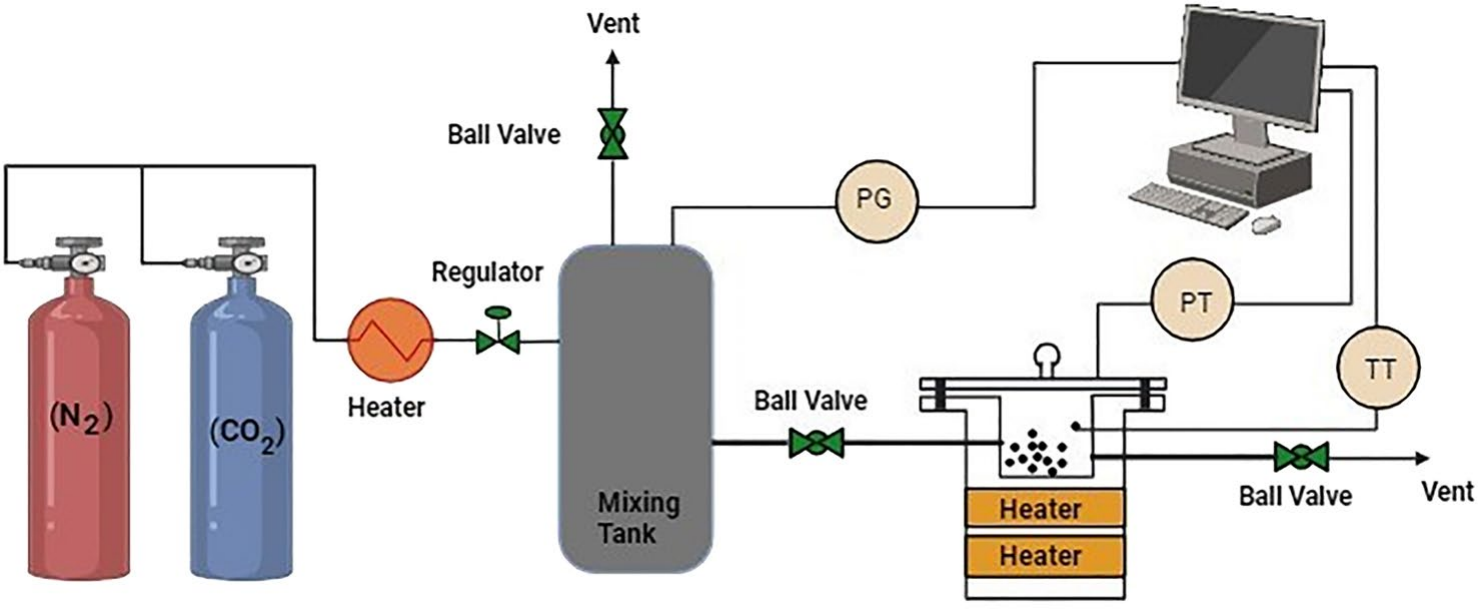
Instrumentation of CO_2_ adsorption setup.

Pure CO_2_ gas was provided through a cylinder, connected with a heater. The pressure and temperature of gas was controlled till it was transferred to reactor containing adsorbent (HCP-AA) for the adsorption experiment. The electrical heater was used to heat the reactor and thermocouple was used to regulate the temperature. Temperature and pressure of CO_2_ were determined at 273 and 298 K by using thermocouple and pressure gauge connected to the computer respectively. Nitrogen gas was used for as an inert medium for discharging and cleaning of mixing tank represented in Fig. 28^71^.

#### Mechanism of adsorption of CO_2_

The HCP-AA showing high BET surface area, abundant pores, carboxyl (-COOH) and amino (–NH_2_) group surged us to investigate their CO_2_ adsorption properties. Nitrogen rich HCPs were used extensively for the CO_2_ adsorption because of polymers host–guest chemistry.

**Figure 29:**
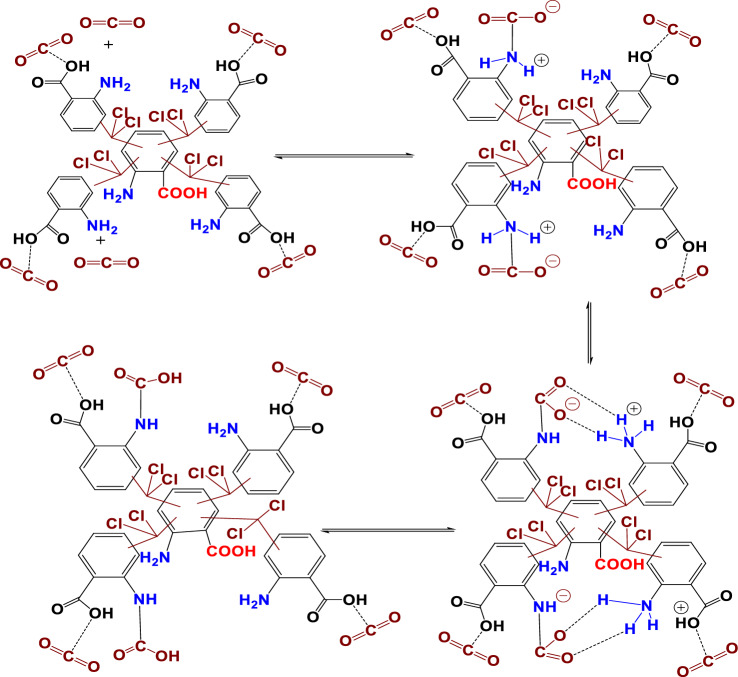
Mechanism of adsorption of CO_2_ on HCP-AA.

There are two ways that HCP-AA can adsorb CO_2_: first, the amine group adsorbed the CO_2_ molecules by the creation of a zwitterion intermediate (R–NH^+2–^COO^−^). A zwitterion intermediate then donates its H^+^ to the nearby amine group to generate ammonium-carbamate ion pairs ((R–NH^3+^–COO–NH–R)), and intermolecular H^+^ transfer can also form carbamic acid (R–NH–COOH) species^72^. General mechanism for the CO_2_ adsorption by HCP-AA through chemisorption is shown in Fig. 29.

#### CO_2_ adsorption

**Figure 30:**
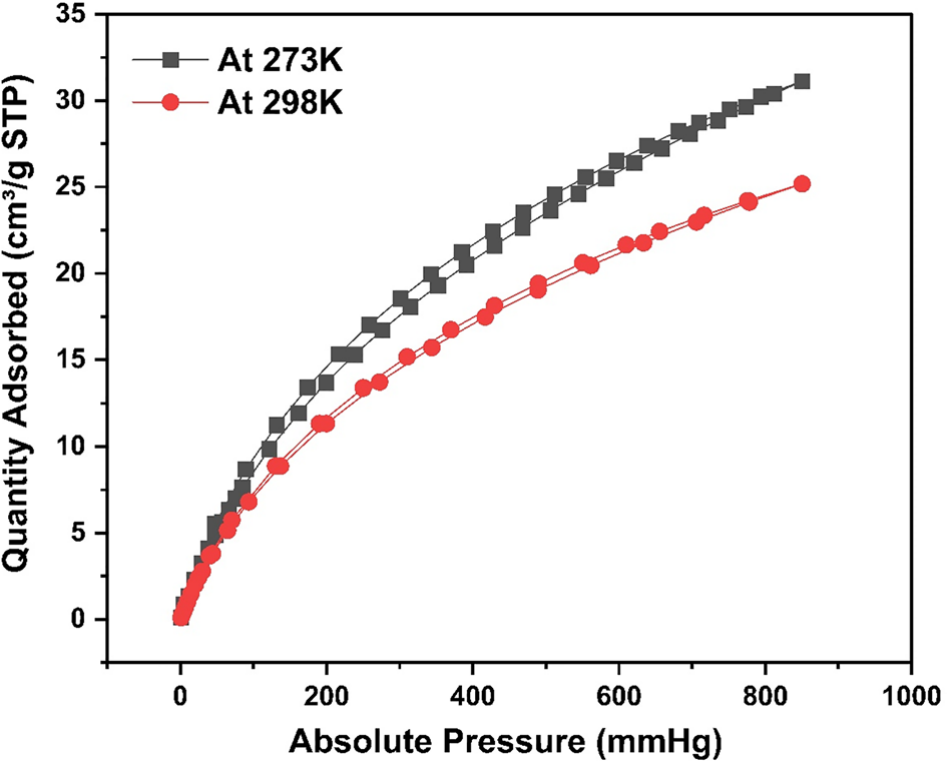
Effect of pressure on quantity of CO_2_ adsorbed (cm^3^/g).

A CO_2_ adsorption study of HCP-AA were conducted at 273 and 298 K is shown in Fig. 30. The adsorption isotherms demonstrating the HCP-AA ability to the CO_2_ uptake at various temperatures.

Quantity of CO_2_ adsorbed on surface of HCP-AA is directly related to pressure applied. As, we increased pressure, the quantity of CO_2_ adsorbed increased as shown in Fig. 30. HCP-AA adsorbed 31.1 cm^3^/g CO_2_ at pressure 850 mmHg at 273 K but at temperature 298 K, it adsorbed 25.2 cm^3^/g at 850 mmHg. The adsorption capacity of the HCP-AA for CO_2_ was a 1.39 mmol/g at 273 K and 1.12 mmol/g at 298 K, which is calculated by using a following relation.

18 $${\text{Adsorption capacity }} = {\text{ moles of CO}}_{{2}} {\text{adsorbed}}/{\text{mass of adsorbent in grams}}\; \times \;1000$$

**Figure 31:**
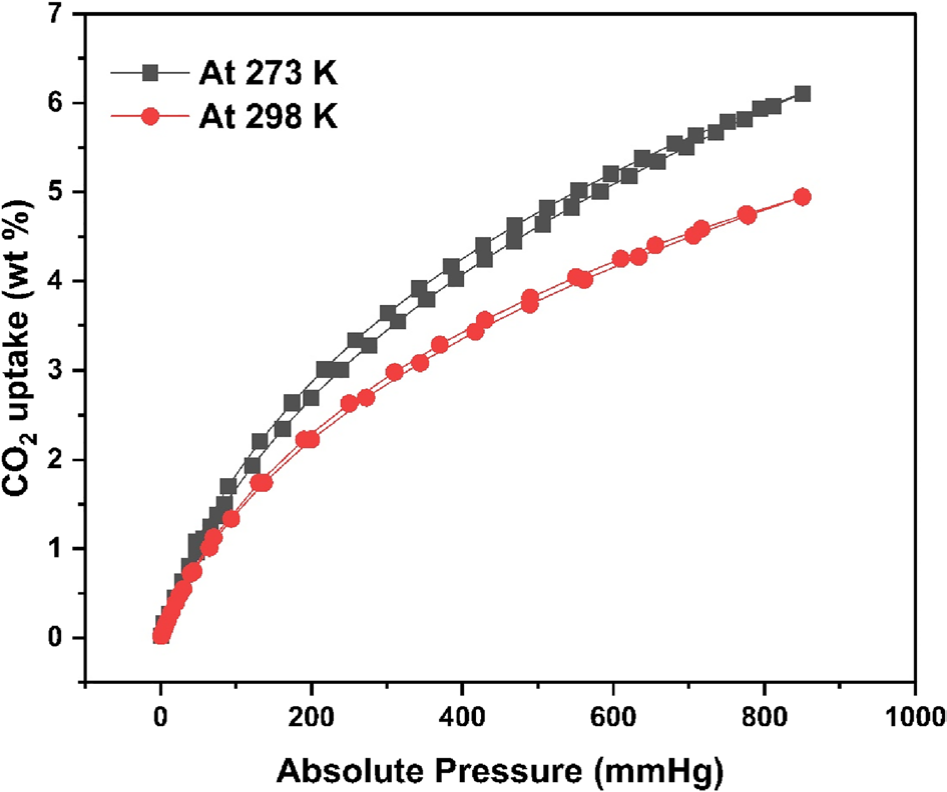
Effect of pressure on CO_2_ uptake (wt%).

It showed a maximum CO_2_ uptake ability of 6.1 wt% at 273 K and 5 wt% at 298 K was because of its high surface area, abundant amino and the carboxyl groups as shown in Fig. 31. The CO_2_ uptake ability reduced at higher temperatures.

**Figure 32:**
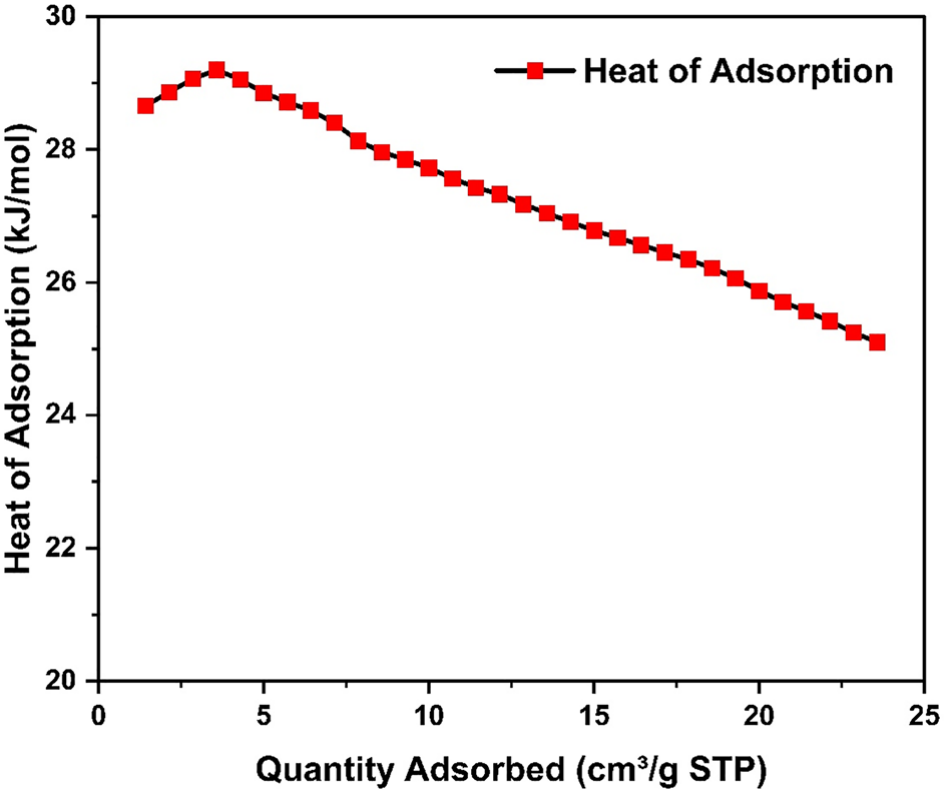
Heat of adsorption of HCP-AA for different quantities of CO_2_.

To clarify the interaction of HCP-AA with CO_2_ molecule, the isosteric heat was estimated by using Clausius–Clapeyron equation. The Q_st_ of the HCP-AA (29.2–25.4 kJ mol^−1^) which is shown in Fig. 32 is because of tightly packed porous structure and nitrogen content.

A comparison of CO_2_ uptake capacity of HCP-AA with other similar adsorbents at 1 bar pressure is shown in Table 10. It shows the adsorption capacity of 1.39 and 1.12 mmol/g at 273 K and 298 K, respectively, which is due to the high surface area, porous surface and the nitrogen content.

**Table 10:** Comparison of HCP-AA performance with other adsorbents.

Adsorbents	CO_2_ uptake capacity (mmol/g)	Temperature (K)	Pressure (bar)	References
HPIM-1	1.73	298	1	^73^
KFUPM-1	1.04	298	1	^74^
HCP-MAAM-2	1.35	273	1	^50^
HCP-MAAM-3	1.28	273	1	^50^
y-POP	1.34	273	1	^75^
KFUPM-2	1.04	298	1	^76^
TPAC-HCP-4	0.9	273	1	^47^
TPE-CPOP1	0.89	298	1	^77^
man-Azo-P1	1.43	273	1	^78^
HCP-PN-1	1.63	273	1	^79^
HCP-PN-1	1.31	298	1	^79^
HCP-PN-2	1.11	273	1	^79^
HCP-PN-2	0.83	298	1	^79^
HCP-AA	1.39	273	1	This study
HCP-AA	1.12	298	1	This study

#### Reusability of HCP-AA for Cr^3+^ and CO_2_ uptake

**Figure 33:**
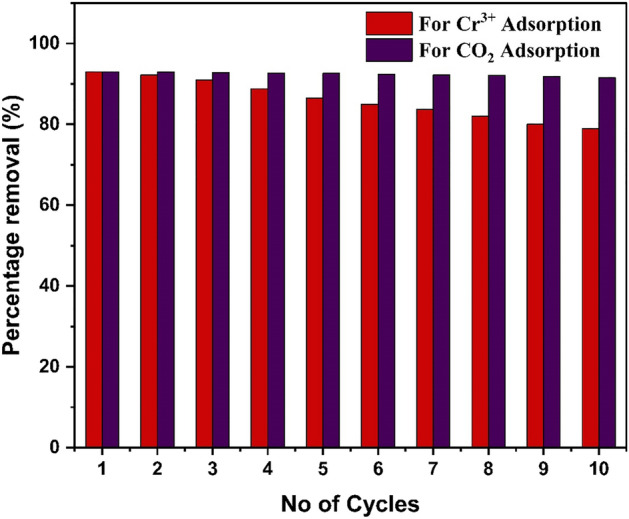
Reusability of HCP-AA for Cr^3+^ and CO_2_ adsorption.

For industrial applications, reusability is the important attribute due to economic value. Conducting repeated adsorption on the same HCP-AA up to 10 cycles reveal that there is a minimal decrease in its efficiency till 3 cycles after that HCP-AA start occupying with heavy metal ions so there is gradually decrease in its efficiency to adsorb further metal ions. For reuse of HCP-AA for several times acidic medium is provided to the HCP-AA by treated it with 0.1 molar HCl solution so desorption takes place but this can regenerate the capability of HCP-AA for some extent. The graph of effect of repeated adsorption experiment on adsorption is shown in Fig. 33.To study the adsorbents recyclability for CO_2_ uptake, the ten adsorption cycles were performed at the 298 K and HCP-AA were recycled for 8 h at 410 K in a vacuum oven. The HCP-AA adsorption potential reduced by 1.5% after 10 cycles.

#### Conclusions and future perspectives

There is no life without plenty of fresh water and air, but due to industrialization and population bloom, clean sources of water are declining day-by-day and air is polluted with greenhouse gases like CO_2_, which can result in global warming. The situation is getting worse in developing countries with few reservoirs of fresh water. One of the main cause of water pollution are heavy metals, which may cause fatal diseases in humans. Therefore, it is an ultimate need to purify water resources. HCPs are excellent candidates to clean water and air through their adsorption capacities as per their structural features like porosity and high surface area. In this article, a simple approach for the synthesis of hyper cross-linked polymer (HCP-AA) through Friedel–Crafts reaction and its use for sequestration of CO_2_ and Cr^3+^ metal ions with potent results are reported. The produced HCP-AA contains oxygen and nitrogen, which gives them a great selectivity and high adsorption capacity for the pollutants along with high stability and reusability. The designed HCP-AA can be a good candidate to solve today’s world problems like global warming and water scarcity. In near future, HCPs have the potential to use in industries and powerhouses, where significant amount of heavy metals, dyes and CO_2_ are emitted. In order to keep environment clean and safe it is better to use such types of HCPs, before the release above-mentioned environmental pollutants to the environment. In order to make it more effective, further work is required to make such HCPs synthesis more feasible, optimized, efficient, and cost effective.

## Data Availability

The datasets used and analyzed in this research are available from corresponding author upon request.
